# Uncovering the connection between obesity and thyroid cancer: the therapeutic potential of adiponectin receptor agonist in the AdipoR2-ULK axis

**DOI:** 10.1038/s41419-024-07084-9

**Published:** 2024-09-30

**Authors:** Changlin Li, Jiao Zhang, Gianlorenzo Dionigi, Nan Liang, Haixia Guan, Hui Sun

**Affiliations:** 1 Division of Thyroid Surgery, the China-Japan Union Hospital of Jilin University, Jilin Provincial Key Laboratory of Surgical Translational Medicine, Jilin Provincial Precision Medicine Laboratory of Molecular Biology and Translational Medicine on Differentiated Thyroid Carcinoma, Changchun City, Jilin Province China; 2 Division of Surgery, Istituto Auxologico Italiano IRCCS, Milan, Italy; 3 Department of Medical Biotechnology and Translational Medicine, University of Milan, Milan, Italy; 4 Department of Endocrinology, Guangdong Provincial People’s Hospital (Guangdong Academy of Medical Sciences), Southern Medical University, Guangzhou, China

**Keywords:** Thyroid diseases, Head and neck cancer

## Abstract

Adiponectin, a unique adipose-derived factor, is significantly downregulated in obesity, making it a crucial target for tumor-related metabolic research. AdipoRon is a novel adiponectin receptor agonist with the advantages of a small molecular weight, high stability and a long half-life. By screening the cervical adipose tissue of papillary thyroid carcinoma (PTC) patients with adipokine antibody array, we found that adiponectin was a potential correlation factor between obesity and PTC progression. AdipoRon has oral activity and is easily absorbed and delivered to target tissues. The effects of AdipoRon on thyroid cancer have not been reported. In this study, we identified adiponectin receptor 1 (AdipoR1) and AdipoR2 on the surface of thyroid cancer cell lines. AdipoRon inhibited the proliferation and migration of thyroid cancer cells, limited energy metabolism in thyroid cancer cells, promoted differentiation of thyroid cancer cells, and induced autophagy and apoptosis. Mechanistic studies revealed that AdipoRon inhibited p-mTOR ^Ser2448^ and p-p70S6K ^Thr389^, and activated ULK1 and p-ULK1. ULK1 knockdown suppressed the effect of AdipoRon on LC3BII/I protein and lysosomes. AdipoR2 knockdown reduced AdipoRon-induced autophagy in thyroid cancer cells. This study is the first to demonstrate the role of AdipoRon in PTC. Our findings illustrate a previously unknown function and mechanism of the AdipoRon-AdipoR2-ULK/*p*-ULK1 axis in PTC and lay the foundation for clinical translation of AdipoRon to PTC. Targeting the AdipoRon-AdipoR2-ULK/*p*-ULK1 axis may represent a new therapeutic strategy for PTC.

## Highlights

- Adiponectin is involved in obesity-related PTC progression.
- AdipoRon inhibits thyroid cancer cell proliferation, migration, energy metabolism, and promotes differentiation.
- The AdipoRon-AdipoR2-ULK/*p*-ULK1 axis is a novel mechanism in obesity-related PTC.
- It offers a potential new therapy and lays the groundwork for clinical translation of AdipoRon.

## Graphical abstract

**Figure:**
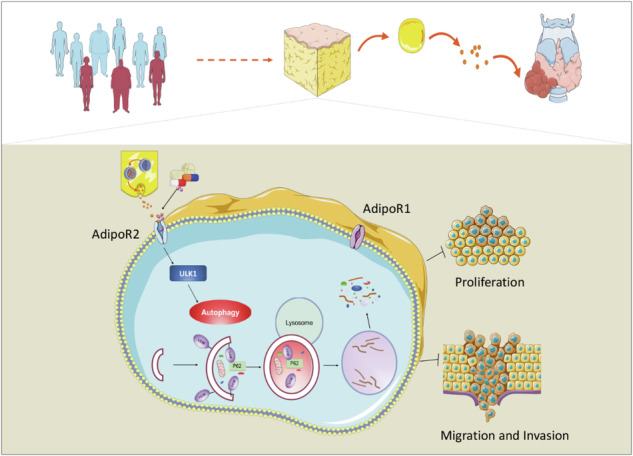
Schematic of AdipoRon induced autophagy in thyroid cancer cell. AdipoRon activates AdipoR2, then activates ULK1 and its phosphorylation site, which induces lysosome formation for autophagy in thyroid cancer cells, affecting proliferation and migration.

## Introduction

Obesity and thyroid cancer are two major challenges in the field of endocrinology and metabolism, and their epidemiological trends are deeply concerning. According to the latest data, the global obese population has exceeded 650 million, and China accounts for a whopping 17%, showing a rapid growth trend [1]. At the same time, the incidence of thyroid cancer is also soaring, with a shocking growth rate of about 13% per year [2]. Among them, papillary thyroid carcinoma (PTC) is the most common pathological type, accounting for 85-90% of thyroid cancer. A large amount of clinical evidence indicates that obesity is closely associated with the risk of PTC occurrence [3, 4]. The clinical characteristics of obese PTC patients are more aggressive. Our retrospective study of 13,995 PTC patients found that obesity increases the risk of lymph node metastasis (OR = 1.387) and invasion(OR = 1.395) [5, 6]. However, the mechanism of obesity promoting PTC progression is still unclear, and there is a lack of targeted intervention measures in clinical treatment. Therefore, it is of great clinical significance to further explore the mechanism of PTC progression associated with obesity and to identify treatment targets.

Adiponectin (AdipoQ) is an adipocyte-derived cytokine encoded by the AdipoQ gene located on chromosome [7]. AdipoQ may be a useful biomarker for the management of metabolic disorders. Low plasma levels of AdipoQ (hypoadiponectinemia) are associated with type 2 diabetes, high plasma levels of triglycerides, low plasma levels of high-density lipoprotein (HDL) cholesterol, hypertension, and coronary dysfunction [7]. AdipoQ may have beneficial effects on insulin resistance, endothelial function, hypertension, ischemic heart disease, atherosclerosis, and oxidative stress [7]. AdipoQ levels are low in patients with breast [8], uterine [9], ovarian [10], prostate [11] cancers, and AdipoQ may halt tumor progression.

The production and use of AdipoQ as a therapeutic option is limited by its complex quaternary structure and rapid turnover. AdipoQ is secreted by adipocytes and circulates as oligomeric complexes, including trimers, hexamers, and high-order structures (12–36 oligomers) of up to 800 kDa [12]. AdipoQ plasma levels are in the microgram per milliliter range, but it has a short plasma half-life of 45–60 min [13]. Purified AdipoQ with four functional regions, including a carboxy-terminal end that may be involved in the formation of a spherical functional domain, can be obtained using an *Escherichia coli* expression system; however, bacterially generated AdipoQ does not possess post-translational modifications.

AdipoQ signals through adiponectin receptor 1 (AdipoR1) and AdipoR2. AdipoRon is a selective, orally active, synthetic small molecule agonist of AdipoR1 and AdipoR2. Adiponectin receptor agonists have potential as therapeutic agents in a variety of diseases [14].

The objective of this study was to investigate the potential role and underlying molecular mechanisms of AdipoRon in papillary thyroid cancer (PTC). To the author’s knowledge, this study is the first to demonstrate a link between an AdipoRon and PTC.

## Methods

### Materials

Materials used in this study, including antibodies, PCR primers, chemicals, peptides, recombinant proteins, commercial assays, software and algorithms are summarized in Supplementary Table 1.

### Cell culture

All cell lines were obtained from the Department of Biochemistry and Molecular Biology, School of Basic Medical Sciences, Tianjin Medical University. Identity of cell lines was verified with Short Tandem Repeat (STR) DNA profiling. HEK293T, K-1, and TPC-1 cell lines were cultured in Dulbecco’s modified Eagle’s medium (DMEM) supplemented with 10% fetal bovine serum (FBS). Nthy-ori 3-1 (normal thyroid follicular cell line; Nthy), BCPAP and KTC-1 (PTC cell lines) cells were cultured in RPMI-1640 with 10% FBS. All cells were maintained in a humidified incubator at 5% CO_2_ and 37 °C.

### Neck adipose tissue adipokine antibody array detection

-- Obtaining neck adipose tissue. Two pieces of adipose tissue at the anterior of the neck (size: 5 mm × 5 mm) were taken, immediately transferred into a sterile tube to avoid drying. They were then placed in a liquid nitrogen container and transported to a −80 °C freezer for storage.

--Experimental specific operation process follows The Quantibody® Human Obesity Array 3 (Catalog Number: QAH-ADI -3, RayBiotech, USA) (Supplementary Table 2). The adipose factor antibody chip includes the following 40 obesity factors, listed in Supplementary Table 3.

### Stable cell lines

Lentivirus was produced by co-transfection of a recombinant lentiviral vector and packaging plasmid into HEK293T cells with Lipofectamine 3000. Virus particles were collected and filtered through a 0.45 μM filter. Stable cell lines were selected using 1 μg/ml puromycin. The efficacy of knockdown for recombinant shRNA vectors was assessed by cloning the recombinant shRNA vectors into pLKO.1-puro. shRNA sequences are listed in Supplementary Table 1.

### High-throughput RNA sequencing (RNA-seq)

Quantity and purity of total RNA were determined using a Bioanalyzer 2100 and RNA 6000 Nano Lab Chip Kit (Agilent, CA, USA). Samples had a RNA Integrity Number (RIN) > 7.0. Sequencing libraries were generated using the NEBNext Ultra II RNA Library Prep Kit for Illumina (NEB, E7760), according to the manufacturer’s instructions. Raw data generated by sequencing were saved in a FASTQ format [15]. The transcriptome was analyzed in a strand-specific manner. The Gene Ontology (GO) database, Kyoto Encyclopedia of Genes and Genomes (KEGG), and Gene Set Enrichment Analysis (GSEA) were used to understand the function of differentially expressed genes and for pathway enrichment analysis. Findings were based on four replicates of each sample.

### RNA extraction and real-time PCR

When cells reached 80-90% confluence, TRIZOL was used to extract RNA. RNA purification was performed using the GeneJET RNA Purification Kit. RNA concentration was determined using a full spectrum microplate reader (Thermo Scientific Multiskan Sky-high). RNA reverse transcription was performed using the RevertAid First Strand cDNA Synthesis Kit. The reaction was incubated at 42 °C for 60 min and heated to 70 °C for 5 min. Real-time PCR used 5 µl SYBR™ Green Master Mix, 0.5 µl each of forward and reverse primers, 1 µl cDNA, and 3 µl H_2_O. See Supplementary Table 4 for a list of primers.

### Western blot and chemical reagents

Western blot was performed using a standard protocol [16]. Whole-cell lysate was prepared using RIPA lysis buffer containing protease and phosphatase inhibitors. Protein concentrations were determined using the Pierce™ BCA Protein Assay Kit. Proteins were separated by sodium dodecyl sulfate-polyacrylamide gel electrophoresis (SDS-PAGE) and transferred to polyvinylidene difluoride (PVDF) membranes. Membranes were blocked in 5% fat-free milk for 1 h and incubated with primary antibodies at 4°C overnight. Secondary antibodies were labeled with horse radish peroxidase (HRP). Signals were detected using an enhanced chemiluminescent Western Blotting Chemiluminescent Substrate kit. ImageJ (http://fiji.sc/Fiji) was used for image visualization, processing and analysis. Antibodies are listed in Supplementary Table 1.

### Cell proliferation and colony formation assays

#### Cell Counting Kit-8 (CCK8)

Cells were counted using a Countess^TM^ 3 (Thermo Fisher Scientific, Freiburg, Germany) automatic cell counter. The mean of three successive counts was used for subsequent experiments. Cell proliferation was evaluated using a Cell Counting Kit-8 (CCK8) viability assay. Cells were seeded (2000 cells/well) in 96-well plates with 100 µL of culture medium. When assessing AdipoRon treatment, the number of cells was adjusted to account for the timing of AdipoRon administration and cell growth rate. Serum-free medium and CCK8 were added to each well at a ratio of 9:1 in the dark. Each assay included a blank control. The plate was premixed and incubated at 37 °C for 1–4 h. Absorbance was measured using a microplate reader at 450 nm against a reference wavelength at 650 nm. Cell growth curves were created with GraphPad Prism 5.

### Clone formation assays

In total, 500–1000 cells were added to a 6-well plate containing 2 ml of medium. Cells were cultured for 5–10 days in a CO_2_ incubator. Cells were fixed, stained with 1% crystal violet, and the number of colonies containing over 50 cells was counted manually.

### EdU assays

EdU (5-ethynyl-2′-deoxyuridine) is a novel thymidine analog. EdU can be incorporated into newly synthesized DNA instead of thymidine. Newly synthesized DNA is labeled with a corresponding fluorescent probe, enabling detection of proliferating cells. 3000 to 8000 cells were cultured on a 24-well plate. A cell proliferation assay was performed using an EdU488 cell proliferation assay kit, according to the manufacturer’s instructions. Cu-induced click chemistry allowed attachment of a fluorescent probe to EdU. EdU488 had an excitation maximum of 495 nm and an emission maximum of 519 nm; Hoechst 33342 (nuclei stained blue) had an excitation maximum of 346 nm and an emission maximum of 460 nm.

### Cell migration assay

#### Scratch wound-healing assay

Cells were cultured to >95% confluence in 6-well plates. Cell-free scratches were 5–10 mm wide. Photographs were taken with an inverted microscope at 0, 6, 12, and 24 h. ImageJ was used to evaluate recolonization of the scratch.

### Transwell assay

In total, 10,000–20,000 cells/200 μl were added to the upper level of a Transwell chamber. Six hundred microliters medium with 10% bovine fetal serum was added to the lower level. Cells were incubated in 5% CO_2_ for 15–24 h. Cells in the lower level were fixed with anhydrous methanol, stained with 1% crystal violet, and photographed. Cells were counted manually.

### Immunofluorescence (IF)

Cells were cultured to 60–70% confluence and fixed in 4% paraformaldehyde at 4 °C for 20 min. Cells were permeabilized with 3% Triton X-100 for 10 min, blocked with 3% BSA for 30 min, and incubated with primary antibodies, including ULK1 (1:300, # 8054, CST) and LC3A/B (1:400, # 2772, CST), at room temperature for 2 h. Cells were incubated with a cross-adsorbed secondary antibody, Alexa Fluor 488, at room temperature for 1 h. Cells were counterstained with 1/1000 Phalloidin Texas Red F-actin at room temperature for 1 h. Cells were stained with DAPI at room temperature for 5 min. Antibodies and other chemical reagents are listed in the Key Resource Supplementary Table 1.

### Oligonucleotide transfection

Three pairs of shRNA sequences targeting exons were designed. Cells were cultured to 60–70% confluence. shRNA was delivered to cells using Lipofectamine 3000, and RNA and protein levels were detected 24 h and 48 h later, respectively. shRNA interfering sequences are listed in Supplementary Table 4.

### mCherry-GFP-LC3B dual-luciferase reporter assay

mCherry-GFP-LC3B dual-luciferase plasmid, lentiviral vector, and the packaging plasmid were co-transfected into HEK293T cells using Lipofectamine 3000. Virus particles were collected and filtered through a 0.45 μM filter. Green fluorescent protein (GFP) and red fluorescent protein (RFP) were detected 24 h after infecting thyroid cancer cells. Cells were fixed, mounted, and photographed.

### Animal experimentation

All animal experiments were conducted in accordance with the Institutional Guidelines for the Care and Use of Animals in Research. This protocol was approved by the Animal Experimental Ethical Inspection Committee of the School of Public Health, Jilin University (NO. SY2024-06-009). Differentiated thyroid cancer cell line KTC-1 (1 × 10^6^ cells per animal) was implanted subcutaneously into 5-week-old male nude mice (BALB/c nu/nu, SiPeiFu, Beijing). Twelve days post-injection, the mice were randomly assigned to eight groups (control group, AdipoRon group, shAdipoR1 (AdipoR1 gene silencing) control group, shAdipoR1 + AdipoRon group, shAdipoR2 (AdipoR2 gene silencing) control group, shAdipoR2 + AdipoRon group, shULK1 (ULK1 gene silencing) control group, shULK1 + AdipoRon group) with five mice in each group. In the AdipoRon group, mice were orally administered 60 mg/kg of AdipoRon every day. In the control group, mice received only PBS solvent as a placebo. Tumor volume was assessed by measuring two perpendicular diameters using a caliper. The formula for calculating tumor volume (*V*) is as follows: *V* = (*a*^2^ × *b*)/2, where ‘*a*’ represents the smaller diameter and ‘*b*’ represents the larger diameter.

### Quantification and statistical analyses

#### Statistical analysis

FPKM values were used to estimate gene expression. log10 (FPKM) value was used to compare expression levels between samples. Cuffdiff software was used to evaluate differential gene expression. Genes that satisfied statistical significance [*q* value < 0.05] were ranked by fold change >1.5 or <0.667 [log2 (1.5) = 0.5849; log2 (0.667) = −0.5849]. GO and KEGG pathway enrichment analysis of differentially expressed genes were implemented with the clusterProfiler package in R (www.r-project.org). Differential genes were defined with GO/KEGG annotations and enrichment was modeled using hypergeometric distribution. A threshold (*p* value) was used to test the enrichment null hypothesis.

Databases were created using Microsoft Office EXCEL (Microsoft Corporation, Redmond, Washington). Measurement data are expressed as mean ± standard deviation; numerical data are expressed as percentage (n/N). Continuous variables were compared with the *t* test or analysis of variance, and categorical variables were compared with the *χ*^2^ test or F’s exact test. Statistical analysis was performed using SPSS 22.0 (windows version) (SPSS, Inc., Chicago, IL, USA) and GraphPad Prism 8.0 software (La Jolla, CA, USC). ImageJ (http://fiji.sc/Fiji) was used for image visualization, processing and analysis. Statistical tests were two-tailed. *P*-values less than 0.05 were considered statistically significant.

## Results

### Screening for potential factors involved in obesity-related PTC progression using adipose factor antibody array

**Fig. 1:**
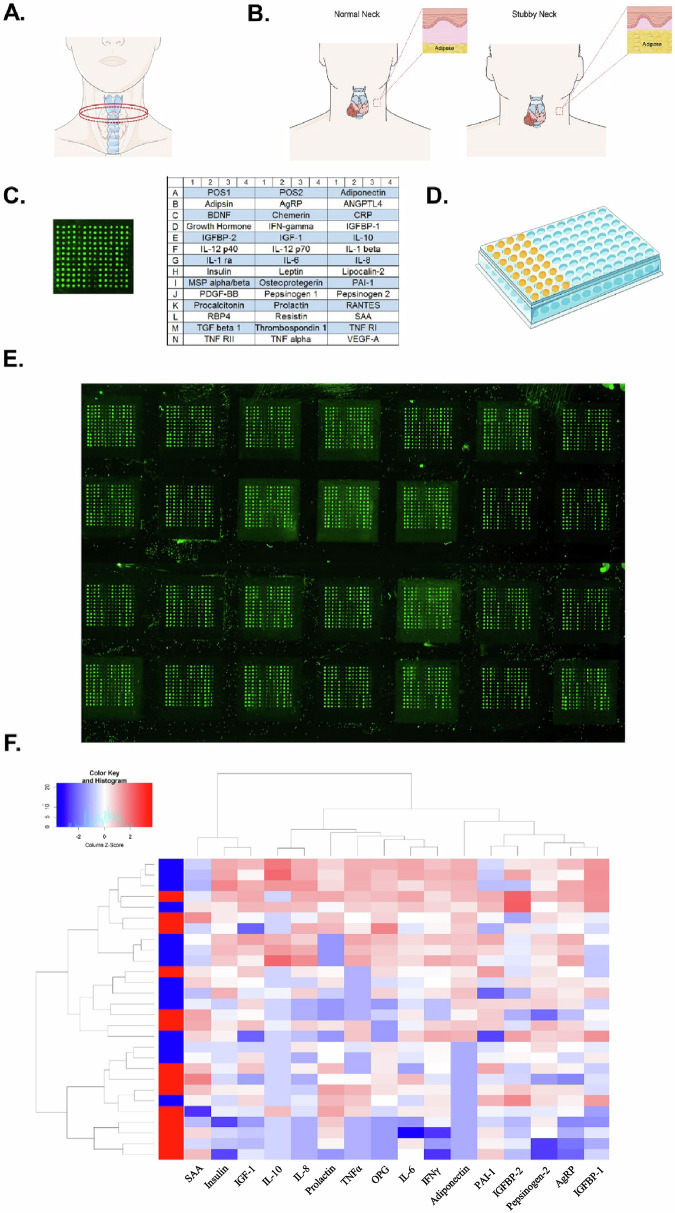
Screening for potential factors involved in obesity-related PTC progression using adipose factor antibody array. **A** The lower edge of the prominent part of the throat and perpendicular to the long axis of the neck, neck circumference. **B** Diagram of subcutaneous fat content in the neck region. **C** The adipokine antibody array layout (40 adipokines). **D** The adipokine antibody array measurement model. **E** Fluorescence scan images of the adipokine antibody array. **F** Hierarchical clustering heatmap of differentially expressed adipokines.

The study prospectively included 28 patients with papillary thyroid cancer (PTC), with 14 patients having normal body mass index (BMI 18.5–24.9 kg/m^2^) and neck circumference, and the other 14 patients being obese in both the whole body and neck, with balanced male-to-female ratio (Supplementary Table 5). To ensure the representative nature of the obese samples, strict inclusion criteria were set: PTC patients with general obesity (BMI ≥ 30 kg/m^2^) [17] and neck obesity (neck circumference > 38 cm for men and > 35 cm for women) [18, 19] (Fig. 1A, B). Subsequently, samples were extracted from the neck adipose tissue of PTC patients, and the expression levels of 40 common adipose factors secreted by adipose tissue were detected using adipose factor antibody chip technology (Fig. 1C–E). The aim was to screen out potential factors related to the progression of obesity-associated PTC. Through the screening and analysis of antibody chips, it was found that adiponectin was significantly lower in obese PTC (Fig. 1F). This finding suggests that adiponectin may be involved in the progression of obesity-associated PTC and adiponectin may have inhibitory effects on PTC, and obesity may promote the development of PTC by reducing adiponectin expression. Therefore, we further explored the mechanism of action of adiponectin receptor agonists, AdipoRon in the development of PTC in subsequent studies.

### The expression of AdipoR1 and AdipoR2 in the membranes of thyroid cancer cells

**Fig. 2:**
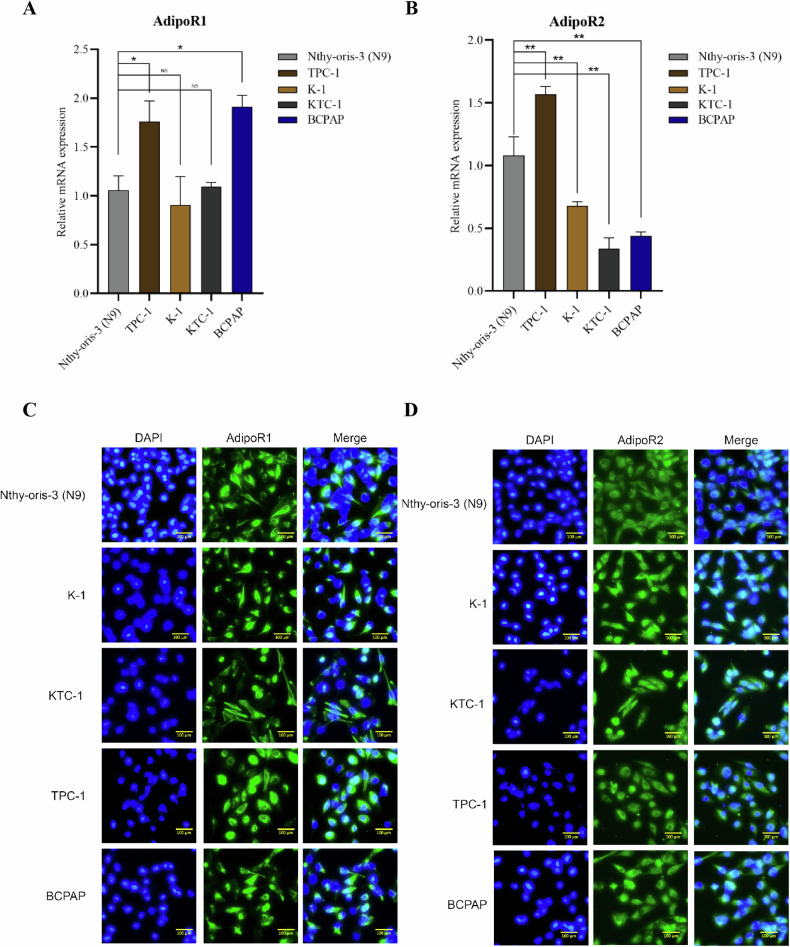
AdipoR1 and AdipoR2 exist on the surface of thyroid cancer cells. **A**, **B** QPCR assay was performed to detect RNA levels of AdipoR1 and AdipoR2 in normal thyroid cells, Nthy-oris-3 (N9), and thyroid cancer cell lines. Data are mean ± SD (*n* = 3); **p < 0.01. **C**, **D** Cells were cultured to 80-90% confluence in 24-well plates. Immunofluorescence staining showed AdipoR1 and AdipoR2 on the surface of thyroid cancer cells. Photographs were taken with a Nikon fluorescent inverted microscope. AdipoR1 (**C**)/AdipoR2 (**D**): green fluorescence; nucleus (DAPI): blue fluorescence. Scale bars, 100 μm.

AdipoR1 and AdipoR2 were expressed in normal thyroid cells, Nthy-oris-3 (N9), and thyroid cancer cells, TPC-1, K-1, KTC-1 and BCPAP. AdipoR2 expression levels were significantly lower in K-1 and KTC-1 cells compared to normal thyroid cells (Fig. 2A, B). Immunofluorescence staining showed AdipoR1 and AdipoR2 on the surface of thyroid cancer cells (Fig. 2C, D).

### AdipoRon inhibits the proliferation of thyroid cancer cells

**Fig. 3:**
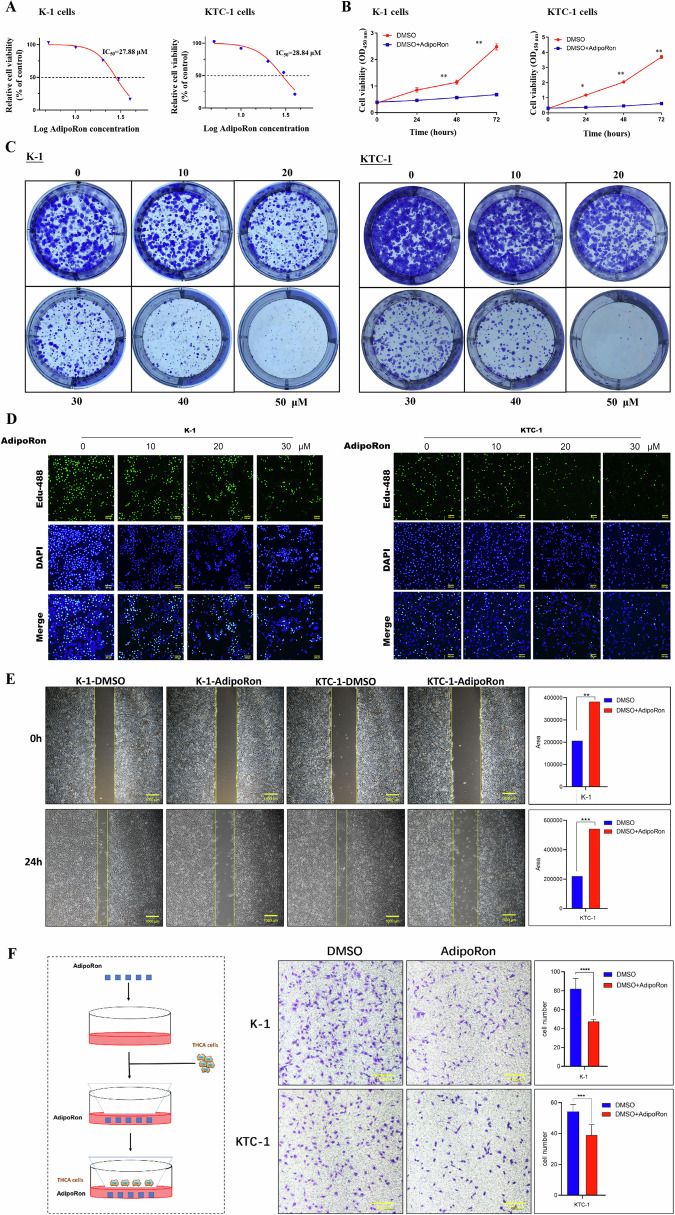
The effect of AdipoRon on the proliferation and migration of thyroid cancer cells. **A** K-1 and KTC-1 cells were treated with increasing concentrations of AdipoRon (0, 10, 20, 30, 40, 50 μM) for 24 h. The CCK8 viability assay was used to detect the IC_50_ of AdipoRon for K-1. Data are mean ± SD (*n* = 6); *p < 0.05, **p < 0.01. **B** K-1 and KTC-1 cells were treated with the IC_50_ of AdipoRon for 0 h, 24 h, 48 h, and 72 h. The CCK8 viability assay was used to measure cell proliferation. Data are mean ± SD (*n* = 6); *p < 0.05, **p < 0.01. **C** Clone formation assays were performed to clarify the effect of AdipoRon on the cloning ability of K-1 and KTC-1 cells treated with increasing concentrations of AdipoRon (0, 10, 20, 30, 40, 50 μM) for 40 h. Cells were fixed and stained with crystal violet. **D** EdU proliferation assays were used to detect the effect of AdipoRon on K-1 and KTC-1 cells. Photographs were taken with a Nikon fluorescent inverted microscope. EdU488: green; excitation maximum 495 nm, emission maximum 519 nm; Hoechst 33342: nuclei stained blue; excitation maximum 346 nm, emission maximum 460 nm. Scale bars, 200μm. **E** Cell scratch assays were performed to determine the effect of AdipoRon on the migratory ability of thyroid cancer cells. K-1 and KTC-1 cells were treated with the IC_50_ of AdipoRon for 24 h. Photographs were taken at 0 and 24 h after scratching. ImageJ was used to evaluate the recolonization of the scratch. Scale bars, 1000μm. Data are mean ± SD (*n* = 3); **p < 0.01, ***p < 0.001**. F** Transwell assays were used to confirm the migratory ability of thyroid cancer cells. K-1 and KTC-1 cells were treated with the IC_50_ of AdipoRon for 24 h. 10,000–15,000 cells were added to the upper layer of the Transwell chamber. Medium containing 10% serum was added to the lower layer. Cells were cultured for 24 h. Cells in the lower layer were stained with 1% crystal violet and photographed under an inverted microscope. The number of migrated cells was counted. Scale bars, 1000μm. Data are mean ± SD (*n* = 3); **p < 0.01, ***p < 0.001.

Thyroid cancer cell lines were treated with various concentrations of AdipoRon. The half maximal inhibitory concentration (IC_50_) of AdipoRon for K-1 and KTC-1 cells were 27.88 μM and 28.84 μM, respectively (Fig. 3A). All subsequent experiments were performed using the IC_50_ of AdipoRon to minimize cellular toxicity. K-1 and KTC-1 cells were treated with the IC_50_ of AdipoRon for 0, 24, 48, and 72 h to determine the effect of AdipoRon on the proliferation of thyroid cancer cells. AdipoRon inhibited the proliferation of thyroid cancer cells in a time-dependent manner (Fig. 3B).

Clone formation assays were performed to clarify the effect of AdipoRon on the cloning ability of thyroid cancer cells. K-1 and KTC-1 cells were treated with increasing concentrations of AdipoRon (0, 10, 20, 30, 40, 50 μM) for 7 days. The cloning ability of thyroid cancer cells decreased in a dose-dependent manner (Fig. 3C).

EdU proliferation assays were used to detect replicating DNA in thyroid cancer cells. K-1 and KTC-1 cells were treated with increasing concentrations of AdipoRon (0, 10, 20, 30 μM). Incorporation of EdU into newly synthesized DNA decreased in a dose-dependent manner (Fig. 3D), confirming the inhibitory effect of AdipoRon on the proliferation of thyroid cancer cells.

### AdipoRon inhibits cancer cell migration

Cell scratch assays were performed to determine the effect of AdipoRon on the migratory ability of thyroid cancer cells. K-1 and KTC-1 cells were treated with the IC_50_ of AdipoRon for 24 h. AdipoRon significantly inhibited cell migration (Fig. 3E). Transwell assays confirmed that AdipoRon inhibited the migratory ability of K-1 and KTC-1 cells (Fig. 3F).

### AdipoRon inhibits cell metabolism

**Fig. 4:**
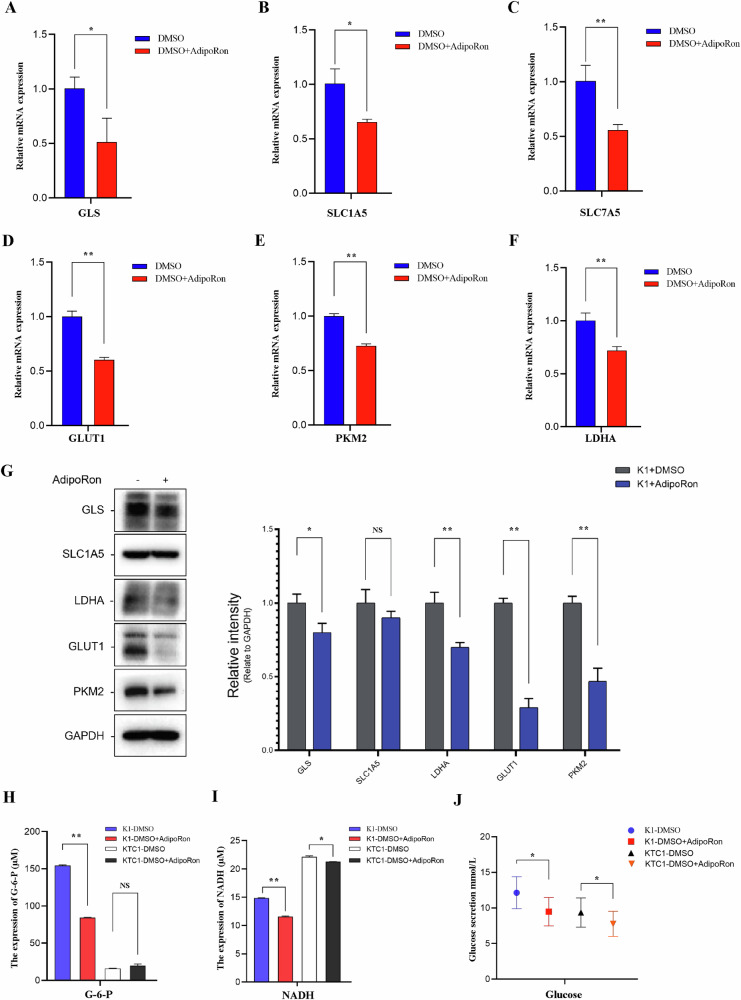
AdipoRon inhibits energy metabolism of thyroid cancer cells. **A**–**F** QPCR was used to detect the RNA levels of key molecules related to amino acid (GLS, SLC1A5, SLC7A5) and glucose (GLUT-1, PKM2, LDHA) metabolism in K-1 cells. Data are mean ± SD (*n* = 3); *p < 0.05, **p < 0.01, ***p < 0.001. **G** Western blot was used to detect protein levels of key molecules related to amino acid and glucose metabolism in K-1 cells. Data are mean ± SD (*n* = 3); NS not significant, *p < 0.05, **p < 0.01. **H** Colorimetric WST-8 assays were used to detect G-6-P levels in K-1 and KTC-1 cells. The maximum absorbance was 450 nm. Data are mean ± SD (*n* = 3); *p < 0.05, **p < 0.01, ***p < 0.001. **I** Colorimetric WST-8 assays were used to detect NADH levels in K-1 and KTC-1 cells. The maximum absorbance was 450 nm. Data are mean ± SD (*n* = 3); *p < 0.05, **p < 0.01, ***p < 0.001. **J** Glucose levels in cell lysates were measured with the o-toluidine method. The maximum absorbance was 630 nm. Absorbance at 630 nm was proportional to the glucose concentration in the sample. Data are mean ± SD (*n* = 3); *p < 0.05.

Metabolism and energy production are important indicators of tumor growth. K-1 cells were treated with the IC_50_ of AdipoRon for 24 h, and RNA and protein levels of key molecules related to amino acid metabolism (GLS, SLC1A5, SLC7A5) and glucose metabolism (GLUT-1, PKM2, LDHA) were detected by qPCR and Western blot. RNA levels of key molecules related to amino acid metabolism (GLS, SLC1A5, and SLC7A5) and glucose metabolism (GLUT-1, PKM2, LDHA) were significantly decreased in K-1 cells treated with AdipoRon compared to control (Fig. 4A–F). Accordingly, protein levels of GLS, PKM2, GLUT-1, and LDHA were significantly decreased in K-1 cells treated with AdipoRon compared to control (Fig. 4G).

Glucose-6-phosphate (G-6-P) plays a central role in energy metabolism. G-6-P is formed by phosphorylation of glucose catalyzed by hexokinase and is involved in metabolic pathways such as glycolysis and pentose phosphatation. NADH is produced during glycolysis, cellular respiration, and the citric acid cycle and is involved in cell metabolism and energy metabolism. NADH is an important marker for the mitochondrial oxidative respiratory chain. Monitoring the redox state of NADH is the best parameter to reflect mitochondrial function. Colorimetric WST-8 assays showed G-6-P levels and NADH levels were significantly decreased in K-1 and KTC-1 cells treated with AdipoRon for 24 h compared to control (Fig. 4H, I). Measurement of glucose by o-toluidine showed glucose levels were significantly decreased in K-1 and KTC-1 cells treated with AdipoRon for 24 h compared to control (Fig. 4J).

### AdipoRon promotes the differentiation of thyroid cancer cells

**Fig. 5:**
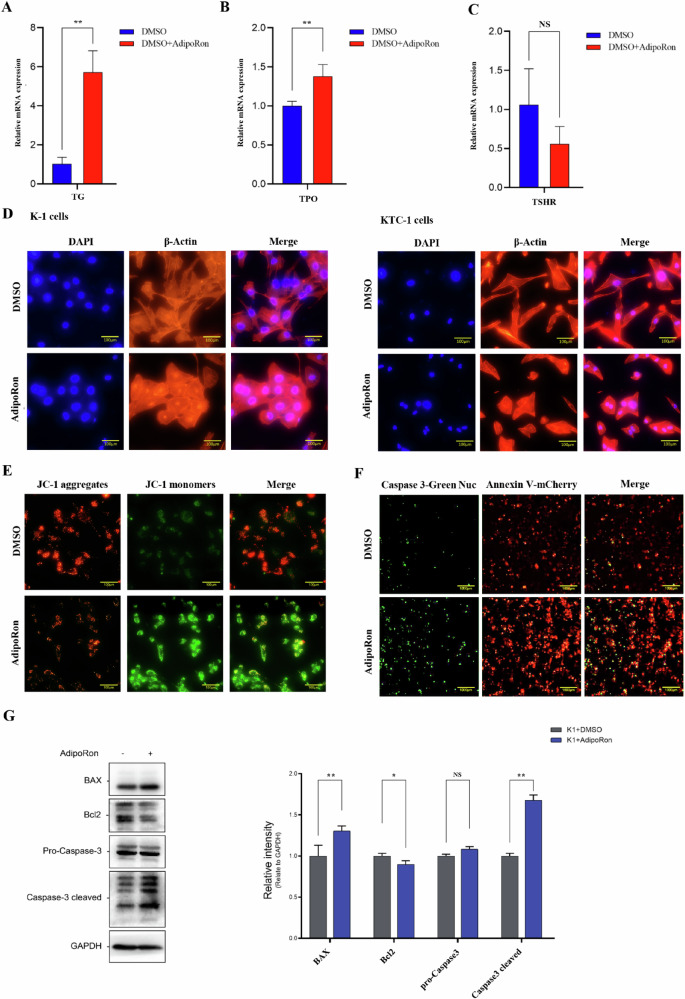
The effect of AdipoRon on the differentiation and apoptosis of thyroid cancer cells. **A**–**C** QPCR was used to detect RNA levels of thyroid-specific proteins, including thyroglobulin (Tg), thyroid peroxidase (TPO) and thyroid stimulating hormone receptor (TSHR), in K-1 cells after AdipoRon treatment. Data are mean ± SD (*n* = 3); *p < 0.05, **p < 0.01. **D** Immunofluorescence staining was used to label the nucleus and cytoskeleton of K-1 and KTC-1 cells. β-actin in the cytoskeleton, red fluorescence; nuclei stained with DAPI, blue fluorescence. **E** Mitochondrial membrane potential of K-1 cells treated with AdipoRon for 40 h. JC-1 was predominantly polymers in the mitochondria of untreated K-1 cells, showing bright red fluorescence and weak green fluorescence. JC-1 was predominantly a monomer in K-1 cells treated with AdipoRon, showing weak red fluorescence and bright green fluorescence. Scale bars, 100 μm. **F** Caspase-3 activity and apoptosis in K-1 cells treated with AdipoRon for 40 h. The nuclei of apoptotic cells with high Caspase-3 activity induced by AdipoRon showed bright green fluorescence, and Annexin V in the cell membrane of apoptotic cells showed red fluorescence. **G** Western blot was used to detect apoptosis-related proteins. Data are mean ± SD (*n* = 3); NS, not significant, *p < 0.05, **p < 0.01.

Abnormal differentiation of tumor cells is a basic biological characteristic of tumors. The detection of thyroid-specific proteins, such as thyroglobulin (Tg), thyroid peroxidase (TPO) and thyroid stimulating hormone receptor (TSHR), may reflect the differentiation ability of thyroid cancer cells. RNA levels of Tg and TPO were significantly increased in K-1 cells treated with AdipoRon for 24 h compared to control (Fig. 5A–C). Immunofluorescence staining was used to label the nuclei and cytoskeleton of thyroid cancer cells. The nuclei of K-1 and KTC-1 cells treated with AdipoRon for 24 h increased in size and the tubules of the cytoskeleton acquired a regular cylindrical shape (Fig. 5D). These data suggest AdipoRon induced differentiation in K-1 and KTC-1 cells.

### AdipoRon promotes apoptosis of thyroid cancer cells

A decrease in mitochondrial membrane potential is a crucial event in the early phase of apoptosis. JC-1 is a fluorescent probe commonly used to detect mitochondrial membrane potential. When mitochondrial membrane potential is high, JC-1 accumulates in the mitochondria as aggregates (JC-1 aggregates). When mitochondrial membrane potential is low, JC-1 is a monomer (JC-1 monomer) and does not aggregate in mitochondria. JC-1 was predominantly a monomer in K-1 cells treated with the IC_50_ of AdipoRon for 40 h, indicating a decrease in mitochondrial membrane potential (Fig. 5E).

The caspase family plays a critical role in controlling apoptosis, with caspase-3 considered a key effector enzyme. Caspase-3 can directly and specifically cleave a variety of substrates, including polyadenosine diphosphate ribose polymerase (PARP), and precursors of caspase-6, caspase-7 and caspase-9. GreenNuc™ caspase-3/7 immunofluorescence was used to detect caspase-3 protein in K-1 cells. Caspase-3/7 protein levels were increased in K-1 cells treated with the IC_50_ of AdipoRon for 40 h compared to control (Fig. 5F).

The exposure of phosphatidylserine on the outer surface of the plasma membrane is an early feature of apoptosis. Annexin V has a high affinity for phosphatidylserine. The expression of Annexin V in K-1 cells was detected using mCherry fluorescently labeled Annexin V (Annexin V-mCherry). Phosphatidylserine levels were increased in K-1 cells treated with the IC_50_ of AdipoRon for 40 h compared to control (Fig. 5F).

Western blot detected proteins associated with apoptosis in K-1 cells treated with the IC_50_ of AdipoRon for 40 h, including BAX, Bcl2, and caspase-3. Bcl2 protein levels were decreased, and BAX and cleaved caspase-3 protein levels were increased in K-1 cells treated AdipoRon compared to control (Fig. 5G). These data suggest AdipoRon induced apoptosis in thyroid cancer cells.

### Transcriptome sequencing of thyroid cancer cells with AdipoRon

**Fig. 6:**
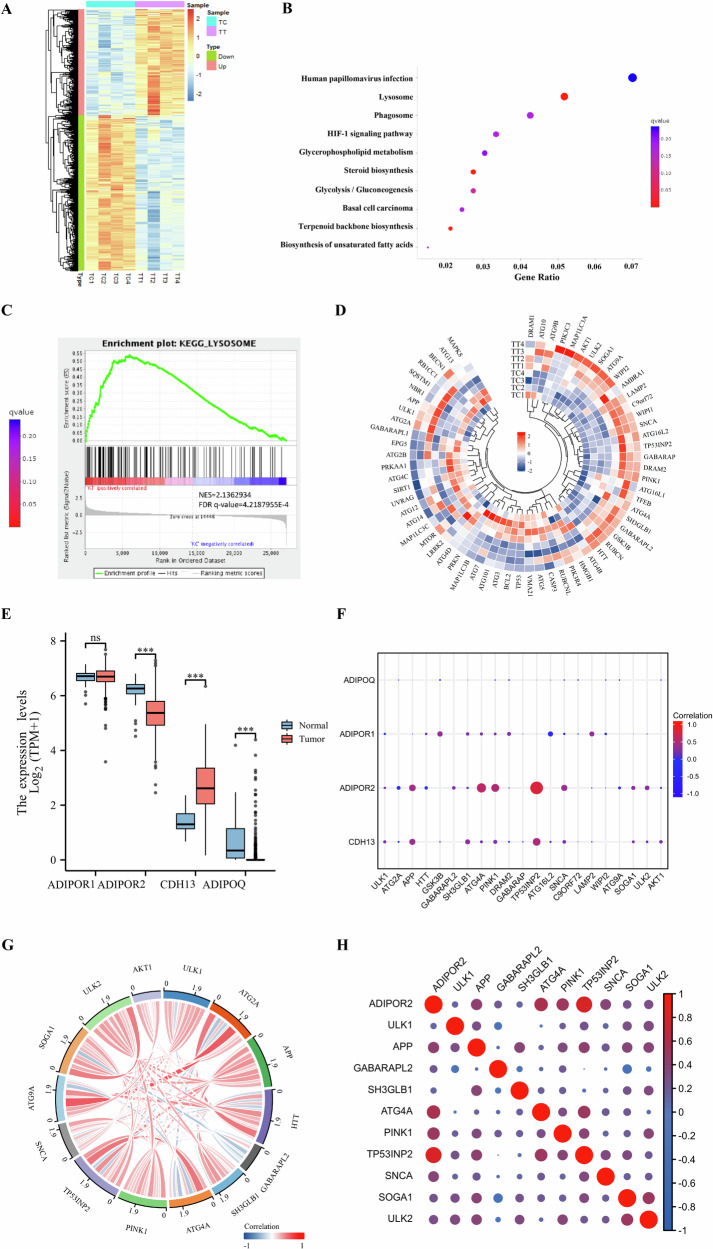
Transcriptome sequencing and bioinformatics analysis of thyroid cancer cells treated with AdipoRon. **A** A heatmap showing gene clustering. **B** KEGG pathway enrichment analysis of transcriptome sequencing. **C** Gene Set Enrichment Analysis (GSEA). **D** Ring-shaped heatmap analysis of autophagy-related genes in thyroid cancer cells after AdipoRon treatment. TT: AdipoRon-treated thyroid cancer cells; TC: control group. **E** Bioinformatics analysis of adiponectin (ADIPOQ) and adiponectin-related receptors in TCGA database. ***p < 0.001. **F** Dot-matrix analysis of the correlation between adiponectin receptor and autophagy differential genes. **G** Chord diagram of differential gene correlations in autophagy. **H** Correlation between adiponectin receptor 2 (ADIPOR2) and autophagy differential genes.

To explore the underlying molecular mechanism by which AdipoRon inhibits the growth of thyroid cancer cells, K-1 cells were treated with the IC_50_ of AdipoRon and the transcriptome was sequenced. There were 5503 differentially expressed genes between K-1 cells treated with AdipoRon and control cells (untreated K-1 cells); of these, 2316 genes were upregulated, and 3187 genes were downregulated (Fig. 6A). KEGG pathway enrichment analysis and gene set enrichment analysis (GSEA) suggested the upregulated genes were involved in lysosome- and phagosome-related pathways (Fig. 6B, C), which are associated with cell death and autophagy. Differential cluster analysis of the genes involved in the autophagy pathway revealed 21 autophagy-related genes were differentially expressed between K-1 cells treated with AdipoRon and control cells (*q* value < 0.05) (Fig. 6D). Bioinformatics analysis of adiponectin (ADIPOQ) and adiponectin-related receptors in TCGA database. Adiponectin receptor 2 (AdipoR2) was significantly downregulated in thyroid cancer tissues (Fig. 6E). AdipoR2 is positively correlated with key autophagy genes, including ULK1, ULK2, ATG4A, PINK1, etc. (Fig. 6F–H).

### AdipoRon induces autophagy in thyroid cancer cells

**Fig. 7:**
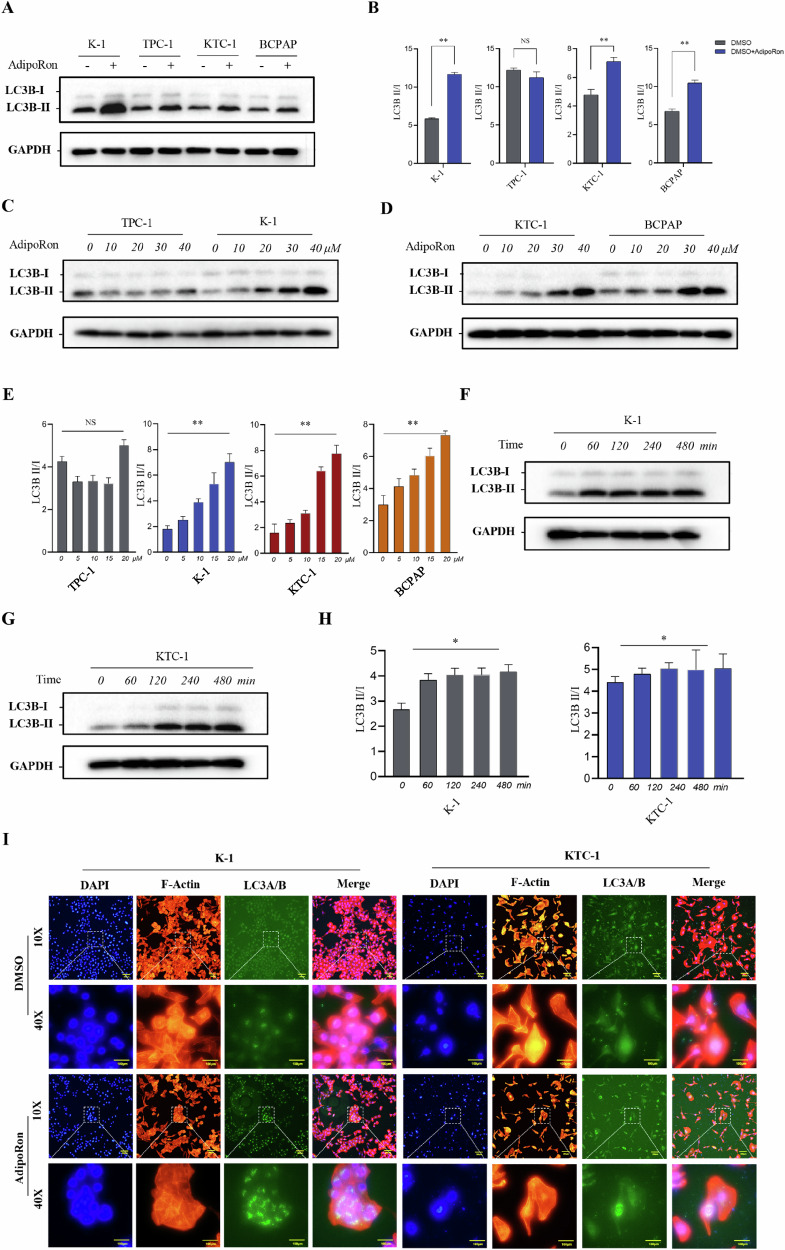
AdipoRon induces autophagy in thyroid cancer cells. **A**, **B** LC3BI/II protein levels after AdipoRon treatment (30 µM for 40 h) in four thyroid cancer cell lines. Data are mean ± SD (*n* = 3); NS not significant, *p < 0.05, **p < 0.01. **C**–**E** AdipoRon increased LC3BII protein level in a dose-dependent manner. Data are mean ± SD (*n* = 3); NS not significant, **p < 0.01; **C** K-1 and TPC-1 cells; **D** KTC-1 and BCPAP cells. **F**–**H** AdipoRon increased LC3BII protein level in a time-dependent manner. Data are mean ± SD (*n* = 3); NS not significant, *p < 0.05; **F** K-1 cells; **G** KTC-1 cells. **I** Immunofluorescence staining detected LC3A/B protein in the cytoplasm of K-1 and KTC-1 cells after AdipoRon treatment (IC_50_ for 40 h). LC3A/B, green fluorescence; nucleus, blue fluorescence; F-actin, red fluorescence. Scale bars, 100 μm.

The molecular mechanism by which AdipoRon induces autophagy in thyroid cancer cells was investigated by detecting changes in the ratio of LC3BII/LC3BI proteins (a key marker of autophagy in mammalian cells) in K-1, TPC-1, KTC-1, and BCPAP cells treated with AdipoRon. The ratio of LC3BII/LC3BI proteins was significantly increased in K-1, TPC-1, KTC-1, and BCPAP cells treated with AdipoRon compared to control, suggesting that AdipoRon can induce autophagy in thyroid cancer cells (Fig. 7A, B). Treatment of K-1, TPC-1, KTC-1, and BCPAP cells with increasing concentrations of AdipoRon (0, 10, 20, 40 μM) for 40 h showed AdipoRon increased the ratio of LC3BII/LC3BI proteins in a dose-dependent manner (Fig. 7C–E). Treatment of K-1 and KTC-1 cells with the IC_50_ of AdipoRon for 0, 60, 120, 240, and 480 min showed AdipoRon increased the ratio of LC3BII/LC3BI proteins in a time-dependent manner (Fig. 7F–H).

**Fig. 8:**
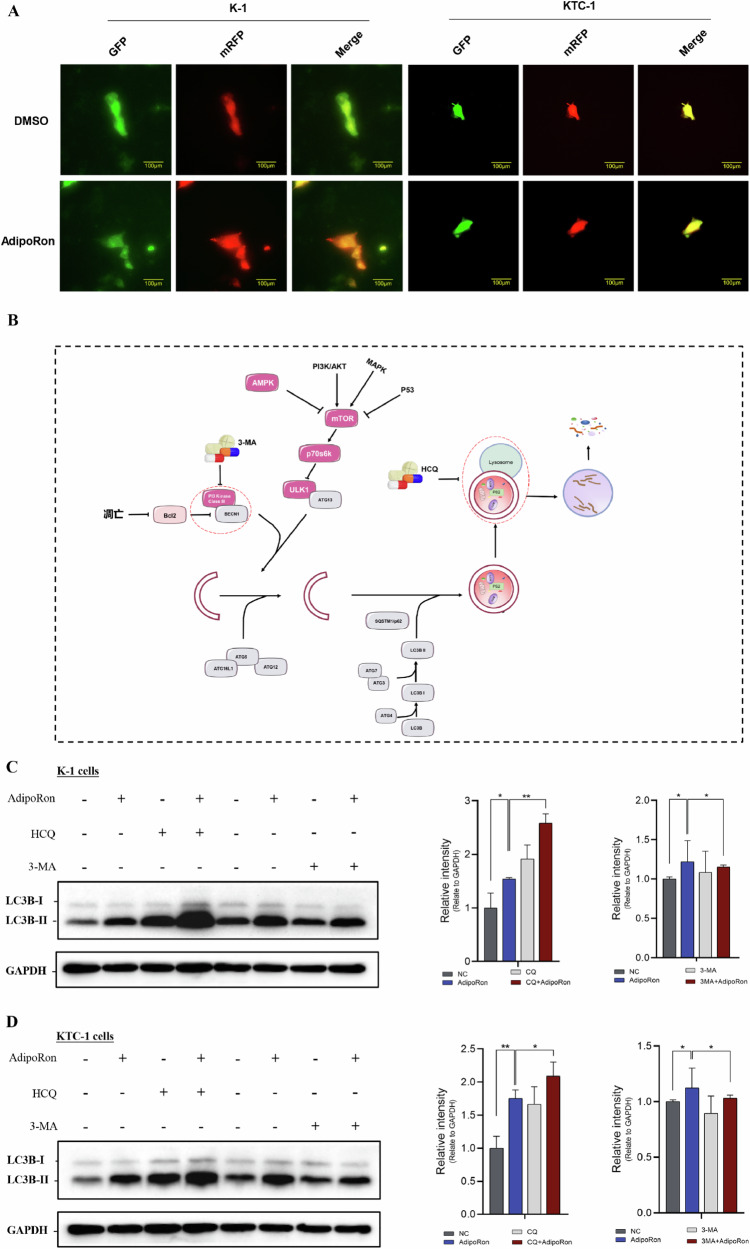
HCQ and 3-MA verify that AdipoRon induces autophagy in thyroid cancer cells. **A** The double-labeled mcherry-GFP-LC3B reporter detected lysosomes involved in autophagy, and showed an increase in lysosomes in K-1 and KTC-1 cells after AdipoRon treatment (IC_50_ for 40 h). In K-1 and KTC-1 cells without autophagy and K-1 and KTC-1 cells containing autophagosomes, co-expression of mCherry and GFP resulted in green fluorescence. When autophagosomes fused with lysosomes to form autophagolysosomes, the acidic lysosomal environment quenched the acid-sensitive GFP, while mCherry was not affected, resulting in red fluorescence. Scale bars, 100 μm. **B** Schematic diagram of the inhibition of the autophagy pathway by HCQ and 3-MA. **C**, **D** Western blot was performed to detect LC3BII/I protein levels in K-1 and KTC-1 cells treated with the IC_50_ of AdipoRon for 40 h and pretreated with HCQ (2 µM, 6 h) or 3MA (6 mM, 6 h). Data are mean ± SD (n = 3); *p < 0.05, **p < 0.01.

Immunofluorescence staining showed an increase in LC3A/B protein in the cytoplasm of K-1 and KTC-1 cells after AdipoRon treatment (IC_50_ for 40 h) compared to control (Fig. 7I). The double-labeled mcherry-GFP-LC3B reporter detected lysosomes involved in autophagy, and showed an increase in lysosomes in K-1 and KTC-1 cells after AdipoRon treatment (IC50 for 40 h) compared to control (Fig. 8A).

### HCQ and 3- MA confirm that AdipoRon induces autophagy in thyroid cancer cells

Hydroxychloroquine (HCQ) and 3-methyladenine (3-MA) are inhibitors of autophagy. HCQ inhibits autophagy by preventing fusion of autophagosomes with lysosomes. 3-MA inhibits autophagy by selectively preventing class PI3K III activity (Fig. 8B). Treatment of K-1 and KTC-1 cells with the IC_50_ of AdipoRon in combination with HCQ or 3-MA was used to clarify that AdipoRon induces autophagy in thyroid cancer cells. The ratio of LC3BII/LC3BI proteins was increased in K-1 and KTC-1 cells treated with AdipoRon alone compared to control. The ratio of LC3BII/LC3BI proteins was further increased in K-1 and KTC-1 cells treated with AdipoRon and HCQ compared to AdipoRon alone. The ratio of LC3BII/LC3BI proteins was decreased in K-1 and KTC-1 cells treated with AdipoRon and 3-MA compared to AdipoRon alone (Fig. 8C, D). These data confirm that AdipoRon induces autophagy.

### AdipoRon activates ULK1 to induce autophagy in thyroid cancer cells

**Fig. 9:**
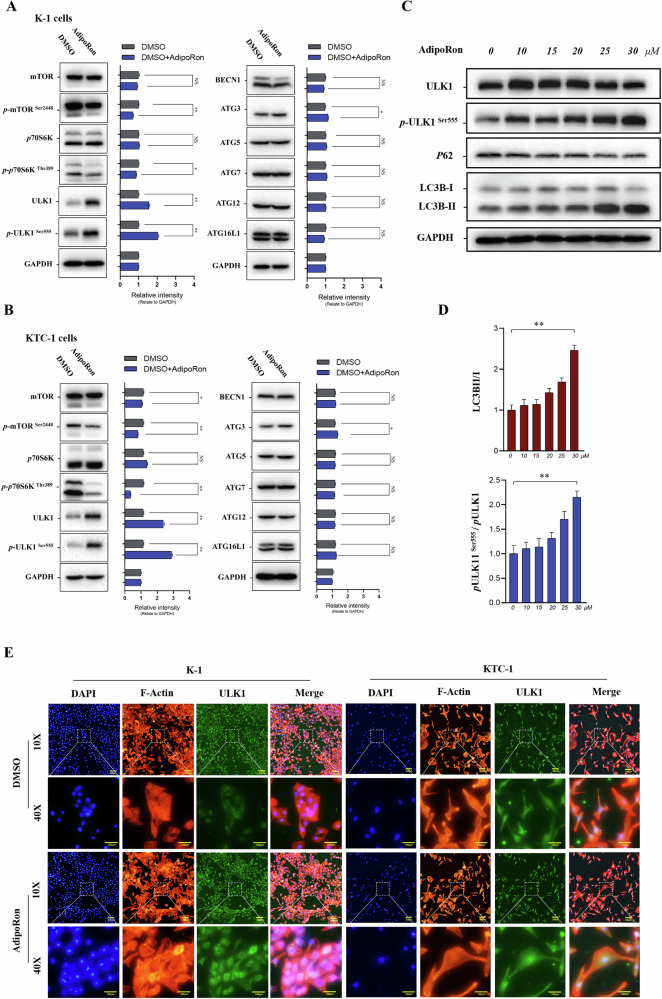
AdipoRon activates ULK1 to induce autophagy in K-1 cells. **A**, **B** Western blot was performed to show changes in autophagy-related protein levels after AdipoRon treatment (IC_50_). Data are mean ± SD (*n* = 3); NS not significant, *p < 0.05, **p < 0.01. **C**, **D** AdipoRon increased p-ULK1 ^Ser555^ protein level in a dose-dependent manner. Data are mean ± SD (*n* = 3); **p < 0.01. **E** Immunofluorescence staining showed ULK1 protein increased in K-1 and KTC-1 cells treated with the IC_50_ of AdipoRon for 40 h. ULK-1, green fluorescence; nucleus, blue fluorescence; F-actin, red fluorescence.

Key proteins of autophagy metabolism and their phosphorylation sites, including mTOR, p-mTOR ^Ser2448^, p70S6K, p-p70S6K ^Thr389^, ULK1, p-ULK1 ^Ser555^, BECN1, ATG3, ATG5, ATG7, ATG12 and ATG16L1, were detected to elucidate the specific molecular mechanisms by which AdipoRon inhibits thyroid cancer cell function. p-mTOR ^Ser2448^ and p-p70S6K ^Thr389^ protein levels were decreased while ULK1 and p-ULK1 ^Ser555^ protein levels were increased in K-1 cells treated with the IC_50_ of AdipoRon for 40 h compared to control (Fig. 9A, B). We conducted bioinformatics analysis to predict potential phosphorylation sites on ULK1. Our analysis revealed that, in addition to Ser555, there was some phosphorylation at Ser638 in response to AdipoRon treatment (Supplementary Fig. 1). Treatment of K-1 cells with increasing concentrations of AdipoRon (0, 10, 15, 20, 25, 30 μM) for 40 h showed AdipoRon increased p-ULK1 ^Ser555^/ULK1 and LC3BII/I protein levels and decreased p62 protein levels in a dose-dependent manner (Fig. 9C, D). We conducted a time-series experiment to observe the temporal changes in ULK1 phosphorylation and autophagy marker proteins following AdipoRon treatment. Our results revealed that phosphorylation of ULK1 preceded changes in LC3BII/I protein levels (Supplementary Fig. 2). Immunofluorescence staining showed ULK1 protein increased in K-1 and KTC-1 cells treated with the IC_50_ of AdipoRon for 40 h compared to control (Fig. 9E).

### Knockdown of ULK1 inhibits AdipoRon-induced autophagy

**Fig. 10:**
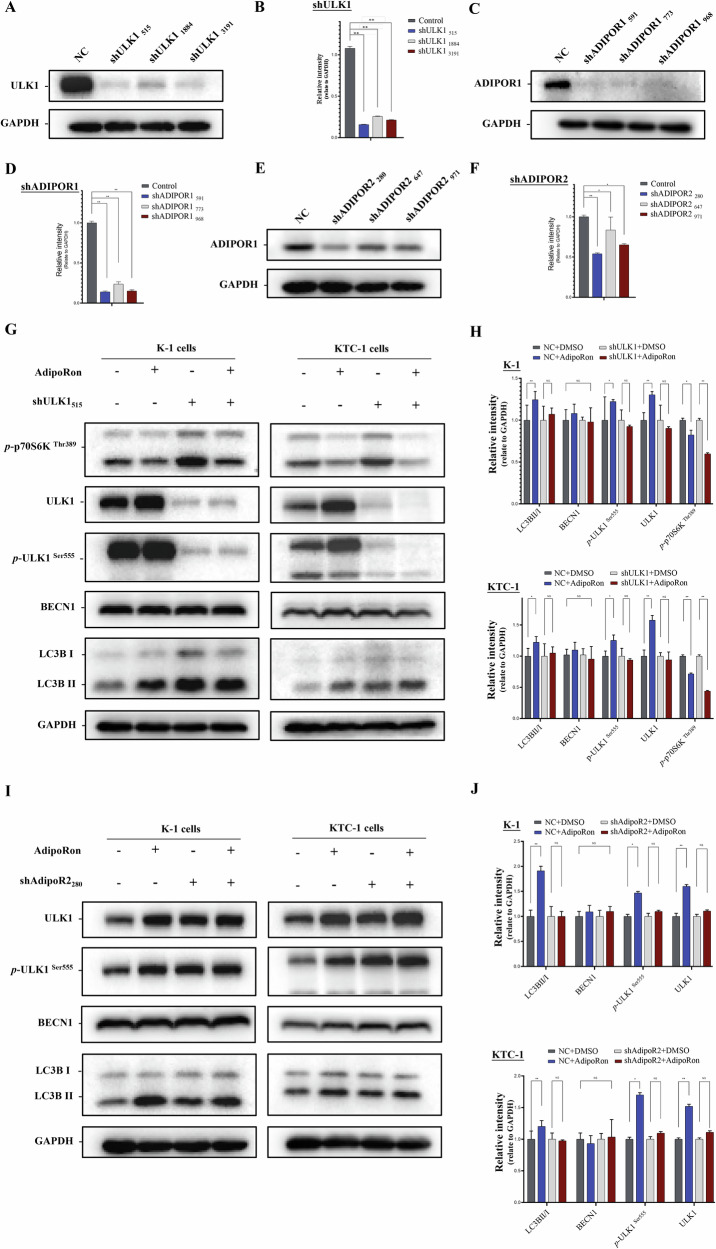
Knockdown of ULK1 and AdipoR2 inhibits AdipoRon-induced autophagy. **A**, **B** Western blot was performed to detect ULK1 protein after knock down by three shRNAs. Data are mean ± SD (*n* = 3); **p < 0.01. **C**, **D** Western blot was performed to detect ADIPOR1 protein after knock down by three shRNAs. Data are mean ± SD (n = 3); **p < 0.01. **E**, **F** Western blot was performed to detect ADIPOR2 protein after knock down by three shRNAs. Data are mean ± SD (*n* = 3); *p < 0.05, **p < 0.01. **G**, **H** AdipoRon promoted the conversion of LC3BI to LC3BII by activating ULK1. Data are mean ± SD (*n* = 3); NS not significant, *p < 0.05, **p < 0.01. **I**, **J** Autophagy-related proteins after AdipoRon treatment and AdipoR2 knockdown. Data are mean ± SD (n = 3); NS not significant, *p < 0.05, **p < 0.01.

**Fig. 11:**
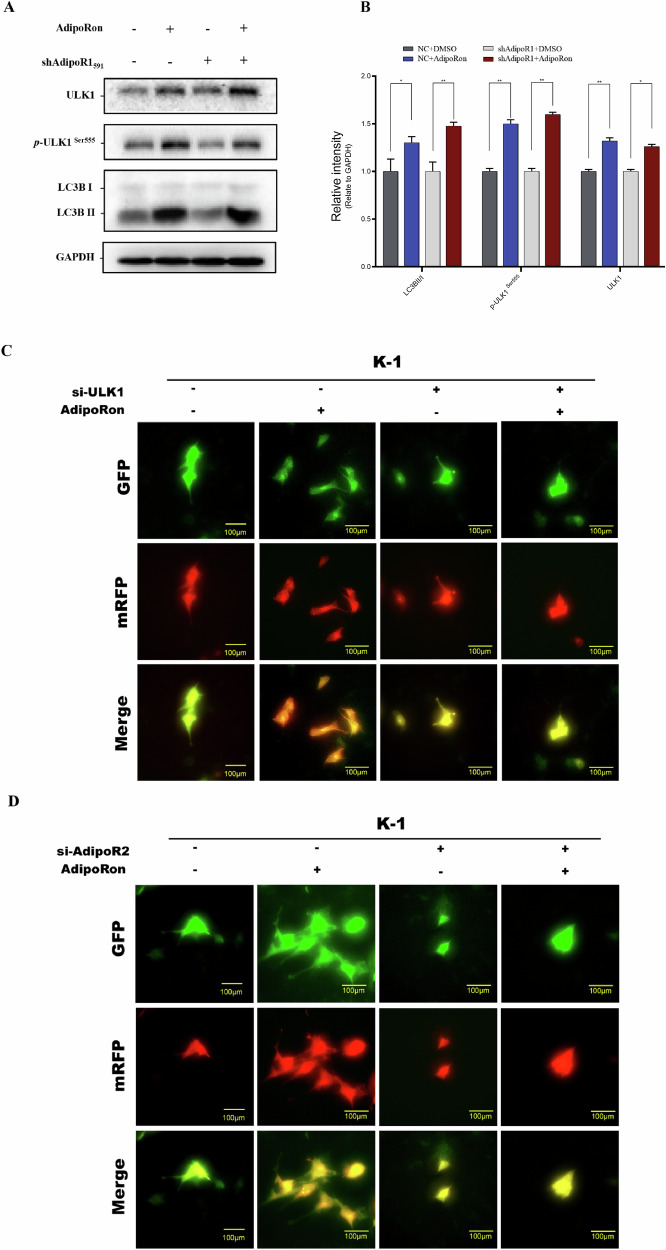
Effects of AdipoRon on autophagy-lysosomal changes in tumor cells. **A**, **B** Autophagy-related proteins after AdipoRon treatment and AdipoR1 knockdown. Data are mean ± SD (*n* = 3); NS, not significant, *p < 0.05, **p < 0.01. **C**. The double-labeled mcherry-GFP-LC3B reporter was used to detect changes in the autophagy-lysosomal system after AdipoRon treatment and ULK1 knockdown. **D**. The double-labeled mcherry-GFP-LC3B reporter was used to detect changes in the autophagy-lysosomal system after AdipoRon treatment and AdipoR2 knockdown.

Three shRNAs targeting the ULK-1 exon sequence were used to clarify that AdipoRon activates ULK1 and its signaling pathway. The shRNA with the highest knockdown efficiency, ULK1-3191, was selected for subsequent experiments (Fig. 10A, B). Phosphorylation of p70S6K ^(Thr389)^, an upstream regulator of ULK1, was decreased in AdipoRon-treated ULK1 deficient K-1 cells compared to AdipoRon-treated wild-type K-1 and KTC-1 cells. Downstream, the ratio of LC3BII/I protein was unchanged in AdipoRon-treated ULK1 deficient K-1 and KTC-1 cells compared to AdipoRon-treated wild-type K-1 and KTC-1 cells (Fig. 10G, H). The double-labeled mcherry-GFP-LC3B reporter showed lysosomes were decreased in AdipoRon-treated ULK1 deficient K-1 and KTC-1 cells compared to AdipoRon-treated wild-type K-1 and KTC-1 cells (Fig. 11C). These data suggest AdipoRon promotes translocation of LC3BI and autophagy by activating ULK1. In vitro, we silenced ULK1 in thyroid cancer cell lines, and used clone formation and scratch assays to demonstrate that AdipoRon regulates tumor cell proliferation and migration capabilities in a ULK1-dependent manner (Supplementary Fig. 3).

### Knockdown of adiponectin receptor 2 (AdipoR2) inhibits activation of ULK1 by AdipoRon

Three shRNAs targeting exons of AdipoR1 or AdipoR2 were used to clarify whether AdipoRon induces autophagy by activating the adiponectin receptor. The shRNA sequence with the highest knockdown efficiency was selected (Fig. 10C–F). pULK1 ^Ser555^ and LC3BII/I protein levels were decreased in AdipoRon-treated AdipoR2 deficient K-1 and KTC-1 cells compared to AdipoRon-treated wild-type K-1 and KTC-1 cells. This suggests that AdipoRon activates ULK1 via AdipoR2 to induce autophagy in thyroid cancer cells (Fig. 10I, J). These data suggest AdipoRon promotes autophagy via AdipoR2. Of note, p-ULK1 ^Ser555^ and LC3BII/I protein levels were increased in AdipoRon-treated AdipoR1 deficient K-1 cells compared to AdipoRon-treated wild-type K-1 cells (Fig. 11A, B). The double-labeled mcherry-GFP-LC3B reporter showed lysosomes were decreased in AdipoRon-treated AdipoR2 deficient K-1 cells compared to AdipoRon-treated wild-type K-1 cells (Fig. 11D).

### AdipoRon inhibits tumor growth of differentiated thyroid cancer cells

**Fig. 12:**
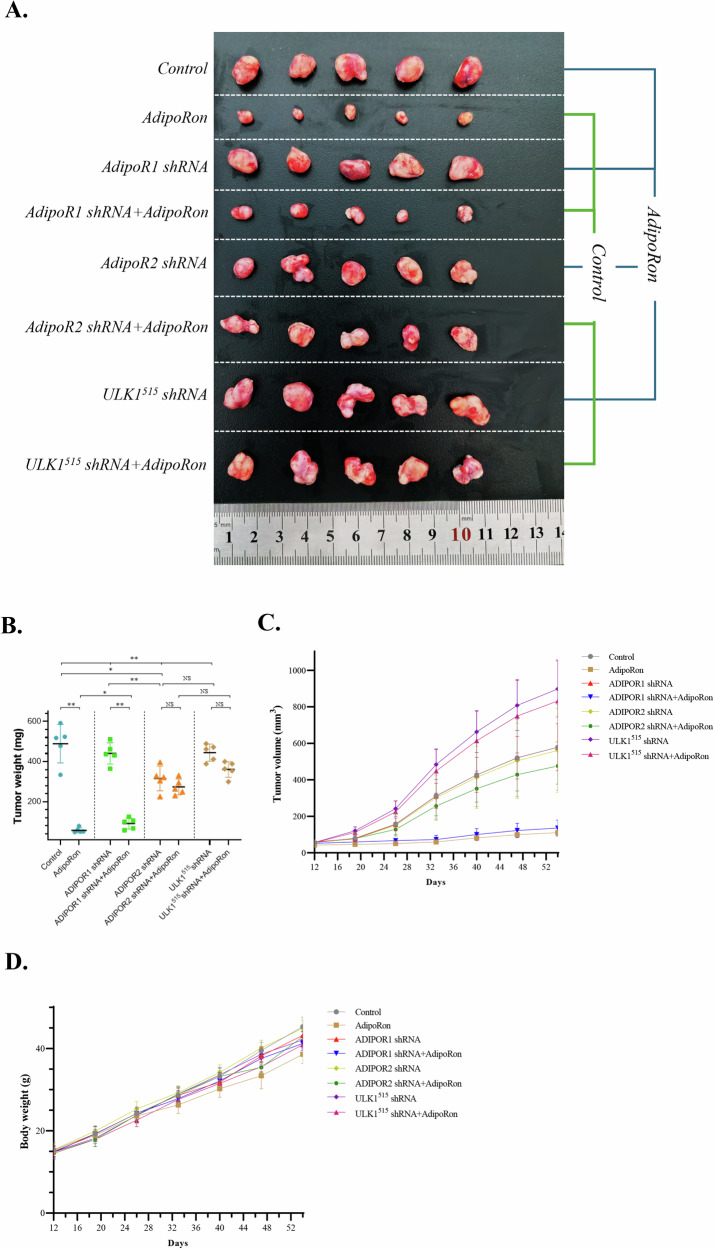
Antitumor Effect of AdipoRon in thyroid cancer. **A** Effect of oral administration of AdipoRon on the growth of subcutaneous KTC-1 tumors. Twelve days after subcutaneous implantation of KTC-1 cells (1 × 10 ^6^ cells), either PBS as a placebo or AdipoRon (60 mg/kg) was orally administered daily (*n* = 5 per group). **B** Tumor weight (n = 5 per group); NS, not significant, *p < 0.05, **p < 0.01. **C** Tumor volume (*n* = 5 per group). **D** Body weight (n = 5 per group).

To mimic the physiological and pathological states of human PTC, we established PTC tumor-bearing mouse models using PTC cell lines with NC (normal control), sh-AdipoR1 (AdipoR1 gene silencing), sh-AdipoR2 (AdipoR2 gene silencing), and sh-ULK1 (ULK1 gene silencing). These models provided us with a platform to investigate the therapeutic effects of AdipoRon under different genetic backgrounds. The mice were treated with AdipoRon through oral gavage (specific experimental methods have been described in detail in the Methods section). The experimental results showed that mice receiving AdipoRon exhibited significant inhibition of PTC growth compared to the control group, manifesting as a reduction in tumor weight (Fig. 12A, B) and tumor volume (Fig. 12C), without any decrement in body weight (Fig. 12D). The inhibitory effect of AdipoRon on tumor growth was significantly attenuated when AdipoR2 and ULK1 were silenced. This suggests that AdipoRon may exert its therapeutic effects by activating AdipoR2 and ULK1 (Fig. 12A–C). However, when AdipoR1 was silenced, AdipoRon was still able to significantly inhibit PTC growth.

### AdipoRon promotes apoptosis of thyroid cancer through autophagy

Thyroid cancer cell lines with silenced ULK-1 or that had been pretreated with an autophagy inhibitor were treated with AdipoRon. A JC-1 fluorescent probe was used as an indicator of mitochondrial membrane potential, as the reduction of mitochondrial membrane potential is a crucial event in the early stage of apoptosis. Findings showed that AdipoRon no longer affected mitochondrial membrane potential in thyroid cancer cells with silenced ULK-1 or that had been pretreated with an autophagy inhibitor (Supplementary Fig. 4). Caspase-3, as a key effector enzyme in the process of apoptosis, was detected in K-1 cells using a GreenNuc™ caspase-3/7 immunofluorescence assay. Findings showed AdipoRon no longer increased Caspase-3/7 protein level in K-1 cells with silenced ULK-1 or that had been pretreated with an autophagy inhibitor (Supplementary Fig. 5). Phosphatidylserine (Annexin V) exposure on the outer surface of the cell membrane is another early feature of apoptosis. Fluorescence detection of intracellular Annexin V exhibited a similar trend to JC-1 (Supplementary Fig. 5).

## Discussion

AdipoQ acts on adipose tissue to increase insulin sensitivity and glucose uptake. Consequently, adiponectin is a pivotal hormone mediating metabolic processes, such as those that involve glucose and triglycerides. AdipoQ has a role in inflammation, especially the immune response, and may exert effects on vascular walls through endothelial and smooth muscle cells. AdipoQ can prevent the formation of free radicals, reduce the synthesis and secretion of C-reactive protein and inhibit TNF-α, a pro-inflammatory mediator. AdipoQ mediates cell growth and apoptosis, and may be an important driver of progression in several cancers. AdipoQ may have a protective effect against colon, lung and pancreatic cancers.

To the authors’ knowledge, this is the first study to investigate the role of AdipoRon in thyroid cancer. We identified AdipoR1 and AdipoR2 on the surface of thyroid cancer cells. In cell function experiments, AdipoRon, a small molecule agonist of AdipoR1 and AdipoR2, inhibited proliferation, clone formation, migration, and invasion in thyroid cancer cells, and induced differentiation in thyroid cancer cells. AdipoRon induced autophagy in thyroid cancer cells via AdipoR2 and by upregulating ULK1.

Visceral adipose tissue that accumulates in the abdomen represents a serious health risk that is associated with obesity, insulin resistance, diabetes, cardiovascular disease, and cancer. Evidence associating obesity and cancer has been reported by numerous studies, which have prompted the introduction of a new term “adiponcosis”, derived from the Latin word “adiposis” (accumulation of fat in the body) and the Greek word “oncosis” (formation of a tumor), to describe the obesity-cancer link.

Obesity is strongly associated with cancers of the colon and rectum, breast (postmenopausal women), endometrium, kidney, esophagus and thyroid. Globally, obesity is estimated to cause 20% of cancers, with ominous prognoses. In the United States, by 2030, more than half the population will be obese, translating to approximately 500,000 new cases of cancer per year.

Obesity and thyroid cancer are common and prevalent diseases, and obesity is an independent risk factor for the development of PTC [3, 4]. We recently conducted a large-scale clinical study in 13,995 adult patients with PTC over a ten-year follow-up. Findings showed that obesity increased the risk of aggressive clinicopathological features of PTC such as extrathyroidal extension and lymph node metastasis [5, 6]. The mechanism by which obesity promotes the progression of PTC is poorly understood, and effective clinical interventions are lacking.

Adipose tissues release a large number of adipokines with anticancer properties that play an important role in tumor growth and metastases. To understand the molecular mechanisms underlying PTC in obese patients, our previous work investigated the role of AdipoQ in the progression of PTC using an AdipoQ-antibody. In vitro functional experiments confirmed that AdipoQ reconstituted from adipose stem cells could significantly inhibit the proliferation and migration of thyroid cancer cells, and that AdipoQ can inhibit the growth and proliferation of thyroid cancer cells by activating AMPK [20].

These data suggest a causal relationship between reduced AdipoQ levels and thyroid cancer in obese patients. Lower than normal AdipoQ levels are associated with several endocrine and metabolic conditions; therefore, weight control may increase AdipoQ levels and reduce thyroid cancer risk. However, only a small number of individuals achieve successful long-term weight loss, suggesting aggressive pharmacological or bariatric surgical interventions may be needed to address the association between obesity and PTC.

Alternatively, ingestion of exogenous AdipoQ may reduce oxidative stress, protect against apoptosis, inhibit leukocyte-endothelial interactions, and reduce smooth muscle proliferation [7]. Circulating levels of AdipoQ are significantly decreased in breast cancer [8], endometrial cancer [9], ovarian cancer [10], prostate cancer [11] and other tumors. However, the clinical use of AdipoQ is limited by its complex quaternary structure, high molecular weight, and short half-life. It is difficult to produce full-length AdipoQ in bacteria, as its collagen amino terminus must undergo post-translational modification in mammalian cells [12]. AdipoQ circulates as higher order structures consisting of trimers, hexamers, and 12–36 oligomers of up to 800 kDa [12]. AdipoQ plasma levels are in the microgram per milliliter range, but it has a short plasma half-life of 45–60 min [13].

AdipoQ exerts its anticancer effects through AdipoR1 and/or AdipoR2. Therefore, the development of a small molecule AdipoQ receptor agonist with low molecular weight, high stability and long half-life has potential as a therapeutic strategy in PTC. In 2013, Okada-Iwabu screened 260,000 compounds and successfully identified a small molecule AdipoQ receptor agonist, AdipoRon, which can activate AdipoR1 and AdipoR2 and exert similar biological functions to AdipoQ [14]. Radioactive binding and Scatchard assays confirmed the specificity of AdipoRon binding to AdipoR1 and AdipoR2 in vitro, with dissociation constants of 1.8 and 3.1 μM [14], respectively. Orally administered AdipoRon is readily absorbed and delivered to the appropriate target tissues, ensuring a treatment effect. Initially, AdipoRon was thought to only have antidiabetic properties. Later, AdipoRon was shown to have anti-obesity, anti-depressant, anti-ischemic, and anti-hypertensive properties [21, 22]. AdipoRon can improve post-traumatic stress disorder, anxiety, Alzheimer’s disease, autoimmune encephalomyelitis, systemic sclerosis, and glomerulonephritis [21, 22]. Recently, AdipoRon was shown to have anticancer properties in several preclinical cancer models, including pancreatic ductal adenocarcinoma, myeloma, and breast, endometrial and ovarian cancer.

Autophagy is an intracellular degradation process that maintains cellular homeostasis by eliminating damaged or redundant organelles and proteins. In various types of tumors, autophagy is generally regarded as a mechanism that promotes tumor growth. RAS activating mutations increase autophagy, thereby enhancing tumor growth, survival, and tumorigenesis, and are associated with mortality due to lung cancer, colon cancer, and pancreatic cancer [23–25]. Therefore, autophagy promotes the survival of tumor cells by enhancing stress tolerance and providing nutrients to meet their metabolic needs, while inhibition of autophagy or knockout of autophagy genes can lead to tumor cell death [26–28]. However, autophagy plays a dual role in both tumor promotion and suppression in cancer biology. Deletion of Beclin 1 has been observed in various human breast, prostate, and ovarian cancers. In mice with knockout of core autophagy proteins, ATG5 and ATG7, the absence of these proteins leads to autophagy-defective hepatocytes that develop liver cancer due to mitochondrial damage and oxidative stress [29].

The role of autophagy in thyroid cancer (TC) is controversial and remains incompletely understood, but there is evidence that it is involved in several key processes. For instance, inhibiting autophagy through drugs can reduce the stemness, migration, and proliferation capabilities of cancer cells, indicating that autophagy may promote TC development [30]. In PTC tissues and cells, the expression of p62 is upregulated, and knocking out p62 can inhibit tumor growth by regulating the AKT/AMPK/mTOR pathway [31].

In some cases, autophagy may exhibit anti-metastatic properties in TC. For example, in PTC and ATC cells carrying BRAF or RAS mutations, autophagy inducers can limit cell proliferation [32]. Similarly, the knockdown or altered expression of certain genes can regulate cell migration, invasion, and apoptosis by affecting autophagy [33, 34]. Additionally, LHPP, a histidine phosphatase with antitumor activity, is downregulated in PTC, and overexpression of LHPP can inhibit AKT/mTOR and activate AMPK signaling, inducing autophagy and reducing cell viability [35, 36],

The relationship between autophagy and apoptosis is also a subject of intense interest. In some cases, autophagy can promote apoptosis, while in others, it may exert a protective effect. For instance, in FTC, PTC, and ATC cell lines, GX15-070 inhibits cell viability by triggering multiple cell death mechanisms, including autophagy. On the other hand, the inhibition of autophagy can enhance or weaken TRAIL-induced apoptosis, depending on the cell type and conditions [37].

Currently, there are few reports on AdipoRon and autophagy. Studies have shown that AdipoRon activates autophagosomes to improve myocardial ischemia-reperfusion (18), promotes epithelial cell autophagy to reduce hypertension-induced epithelial-mesenchymal transition and renal fibrosis [21], and promotes autophagy to reduce chondrocyte calcification in osteoarthritis [22]. To the authors’ knowledge, there are no reports on the association between AdipoRon and autophagy in tumor cells. This study shows that AdipoRon can induce autophagy in thyroid cancer cells via AdipoR2 and upregulating ULK1, thereby inhibiting tumor growth.

Our study observed differences in ULK1 phosphorylation and autophagy marker protein changes in thyroid cancer cells. ULK1 phosphorylation is crucial in autophagy, and AdipoRon activates both ULK1 and its phosphorylated form p-ULK1 Ser555, suggesting it regulates autophagy through ULK1 phosphorylation. Changes in autophagy marker proteins like LC3BII/I reflect autophagy activity. However, autophagy is complex, involving multiple signaling pathways, leading to differences in marker protein changes. Time-series experiments showed that the differences in ULK1 phosphorylation and autophagy marker protein might be partially attributed to the temporal dynamics of autophagy induction and the varying activation patterns of different signaling pathways. Moreover, Our analysis revealed that, in addition to Ser555, there was some phosphorylation at Ser638 in response to AdipoRon treatment. This finding underscores the importance of considering multiple phosphorylation sites on ULK1 when assessing its regulatory role on autophagy. While Ser555 phosphorylation provides a useful indicator of ULK1 phosphorylation, it may not fully capture all phosphorylation events occurring within the protein.

Findings from this study imply that AdipoRon can inhibit tumor growth by inducing autophagy. AdipoRon may represent an effective treatment strategy for obesity-related cancers, supporting the clinical implementation of AdipoRon in PTC.

## Conclusion

AdipoRon is a novel adiponectin receptor agonist. AdipoRon inhibited the proliferation and migration of thyroid cancer cells, limited energy metabolism in thyroid cancer cells, promoted differentiation of thyroid cancer cells, and induced autophagy. Mechanistic studies revealed that AdipoRon activated ULK1 and p-ULK1. ULK-1 knockdown suppressed the effect of AdipoRon on LC3BII/I protein and lysosomes. AdipoR2 knockdown reduced AdipoRon-induced autophagy in thyroid cancer cells. Our findings illustrate that targeting the AdipoRon-AdipoR2-ULK/p-ULK1 axis may represent a new therapeutic strategy for PTC.

## Supplementary information

Supplementary Figures

Supplementary Tables

Original Western blots

## Data Availability

The raw data supporting the conclusions of this article will be made available by the authors, without undue reservation.
