# Supplementary material for: Uncovering the connection between obesity and thyroid cancer: the therapeutic potential of adiponectin receptor agonist in the AdipoR2-ULK axis
Source: Cell Death Dis. 2024 Sep 30;15(9):708. doi: 10.1038/s41419-024-07084-9 (PMC11443080; doi:10.1038/s41419-024-07084-9)
Supplement: Supplementary file 1 — Supplementary Figures [file 41419_2024_7084_MOESM1_ESM.docx]

**Supplementary Figure 1.**


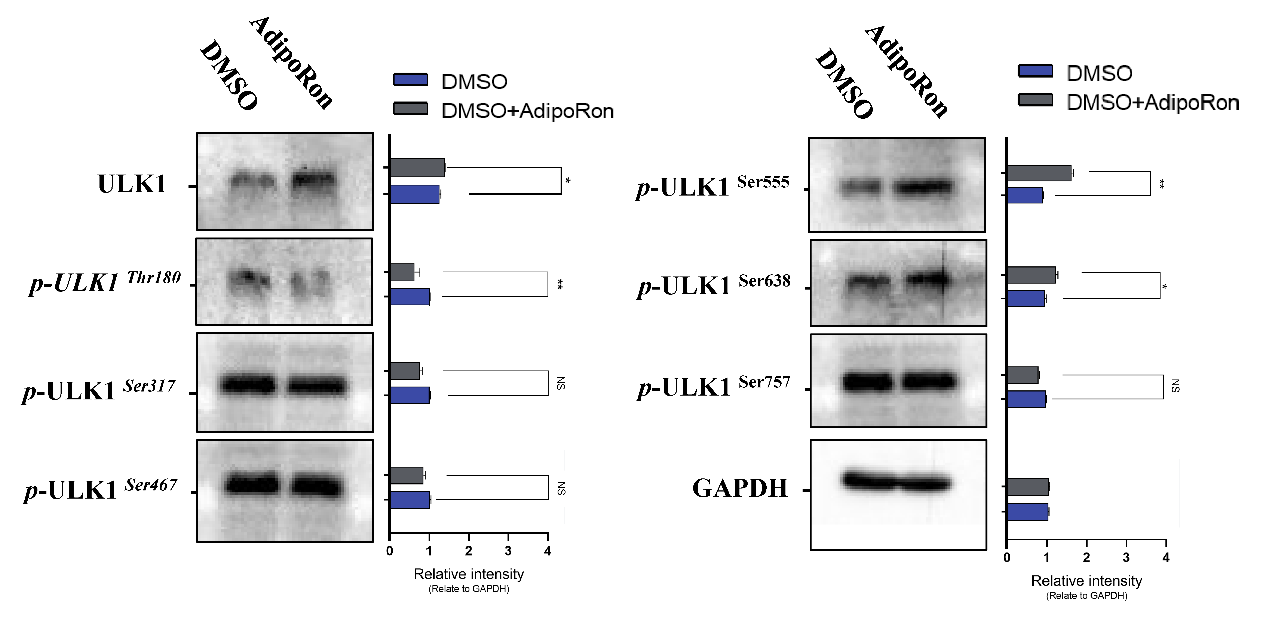


**Supplementary Figure 1. Influence of AdipoRon on phosphorylation sites on ULK1 in PTC cells.**

Data are mean±SD (n=3); NS, not significant, **p*<0.05, ***p*<0.01.

**Supplementary Figure 2.**


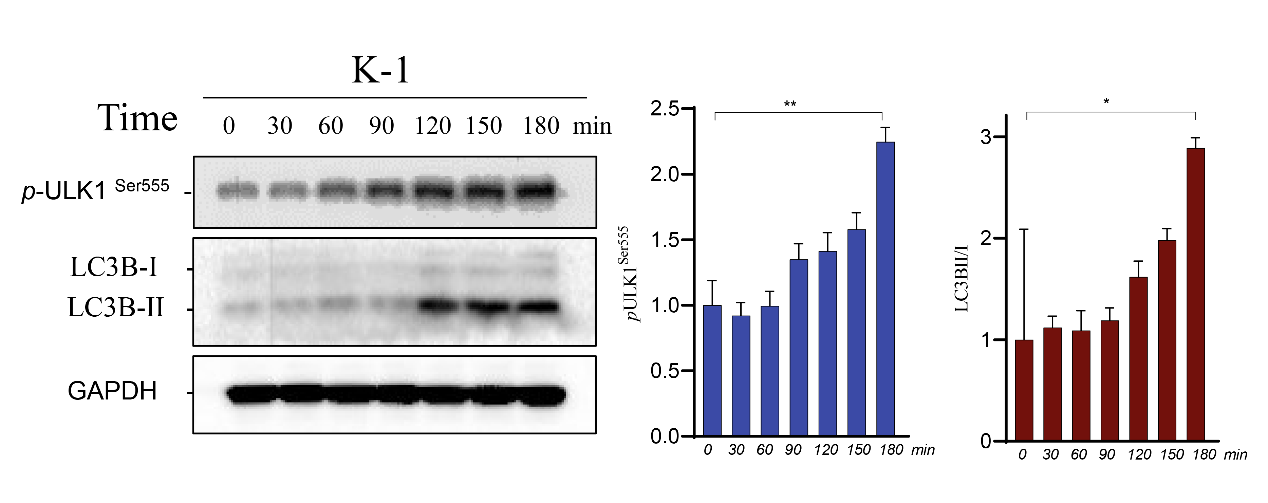


**Supplementary Figure 2. AdipoRon increased ULK1 phosphorylation at Ser 555 (p-ULK1 ^Ser555^) and LC3BII/I protein levels in a time-dependent manner.**

Data are mean±SD (n=3); **p*<0.05, ***p*<0.01.

**Supplementary Figure 3.**


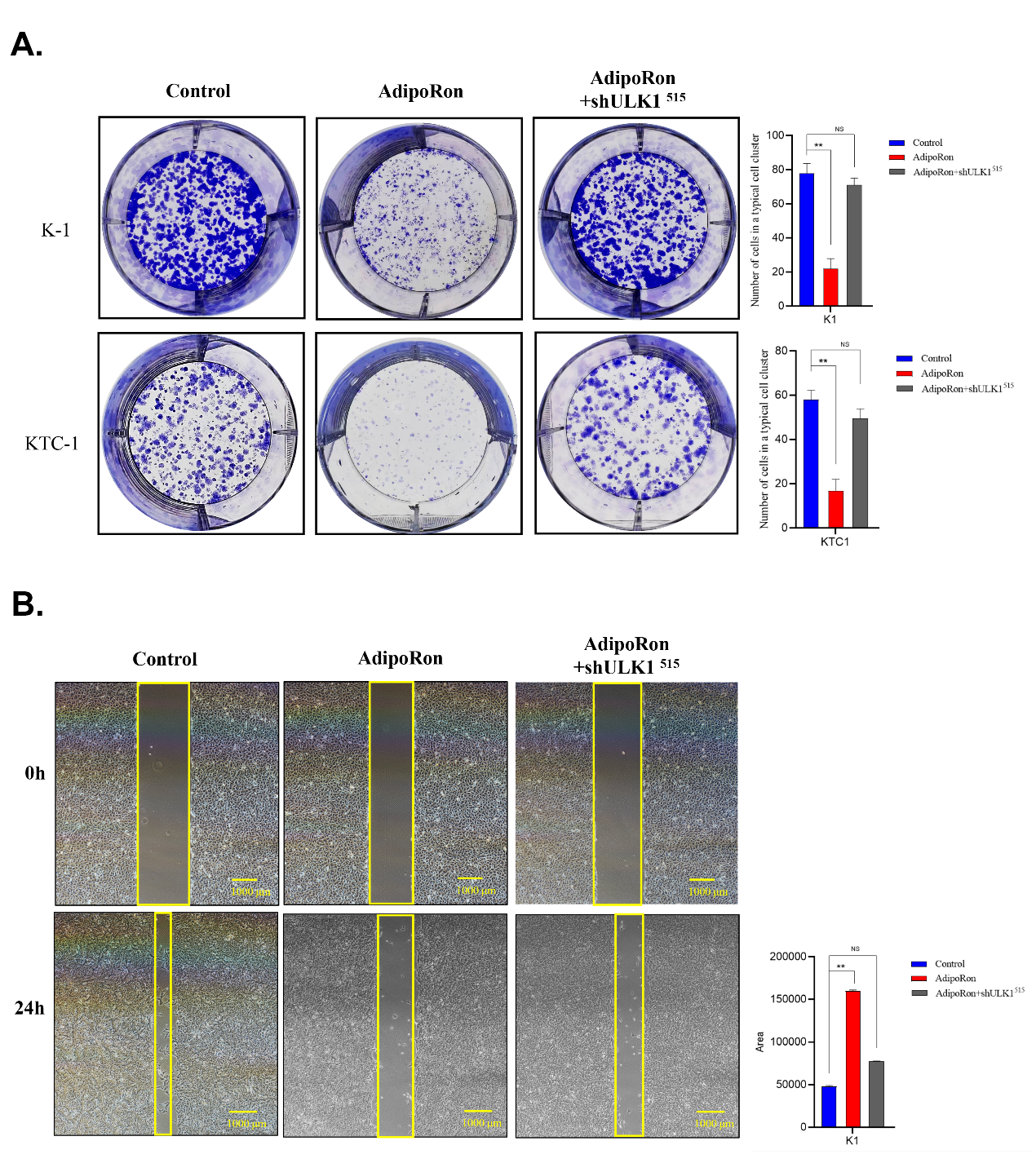


**Supplementary Figure 3. The effect of AdipoRon on the proliferation and migration of shULK1 thyroid cancer cell lines.**

A. Clone formation assays were conducted to elucidate the impact of AdipoRon on the cloning capability of K-1 and KTC-1 cells pretreated with the IC_50_ of AdipoRon for 40 hours with or without shULK1. Cells were fixed and stained with crystal violet. Data are mean±SD (n=3); **p<0.01. B. Cell scratch assays were performed to determine the effect of AdipoRon on the migratory ability of thyroid cancer cells. The IC_50_ of AdipoRon was used to treat cells with or without shULK1 for 24 hours. Photographs were taken at 0h and 24h after scratching. ImageJ was used to evaluate recolonization of the scratch. Scale bars, 1000μm. Data are mean±SD (n=3); **p<0.01.

**Supplementary Figure 4.**


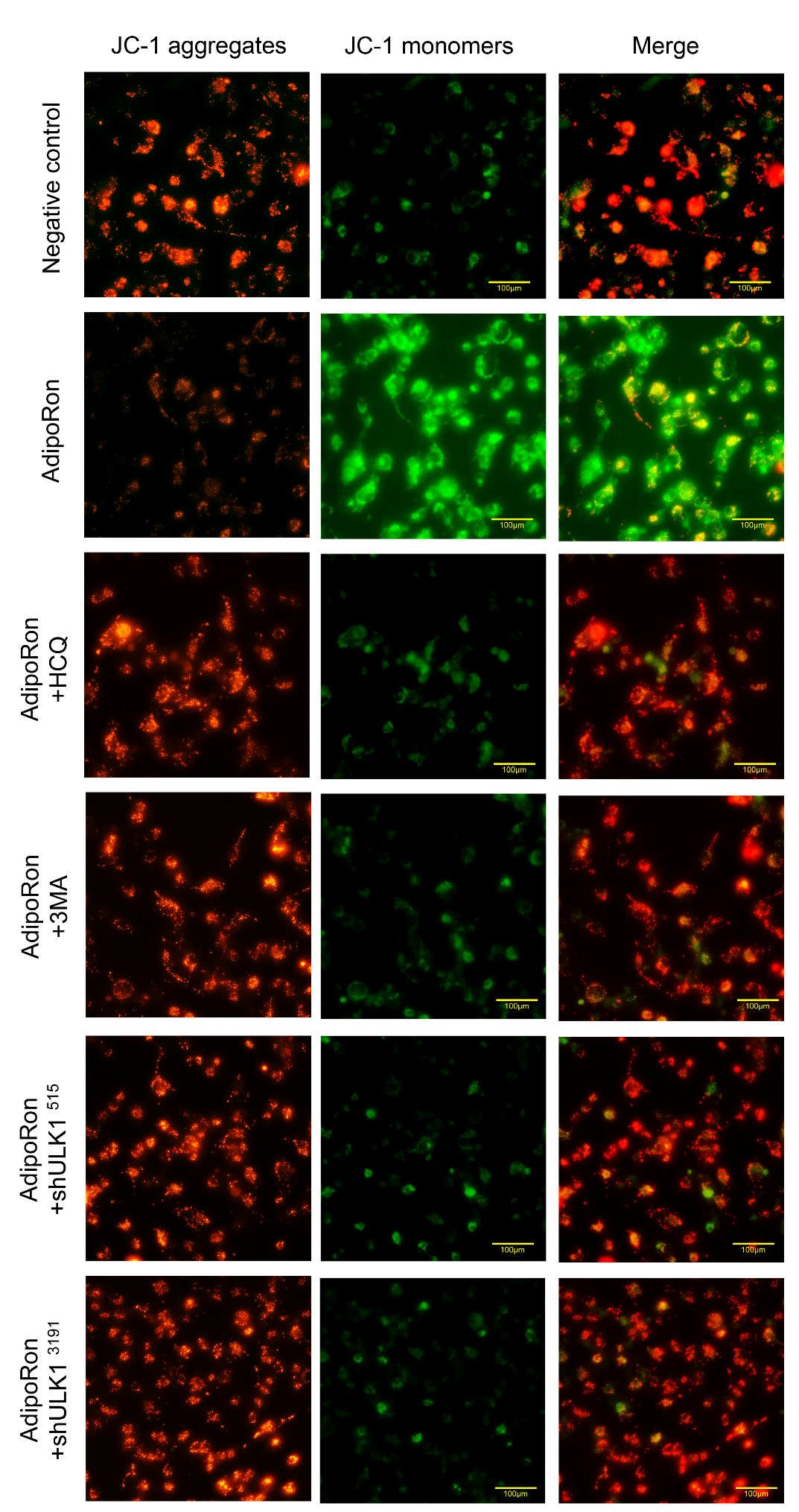


**Supplementary Figure 4. The regulatory role of AdipoRon on mitochondrial membrane potential and autophagy in PTC cells.**

Mitochondrial membrane potential of K-1 cells treated with AdipoRon for 40h (AdipoRon). AdipoRon +3MA K-1 cells were pretreated with 3MA (6 mM, 6 h). AdipoRon+HCQ K-1 cells were pretreated with HCQ (2 µM, 6 h). JC-1 was predominantly polymers in the mitochondria of untreated K-1 cells, showing bright red fluorescence and weak green fluorescence. JC-1 was predominantly monomers in K-1 cells treated with AdipoRon, showing weak red fluorescence and bright green fluorescence. Scale bars, 100μm.

**Supplementary Figure 5.**


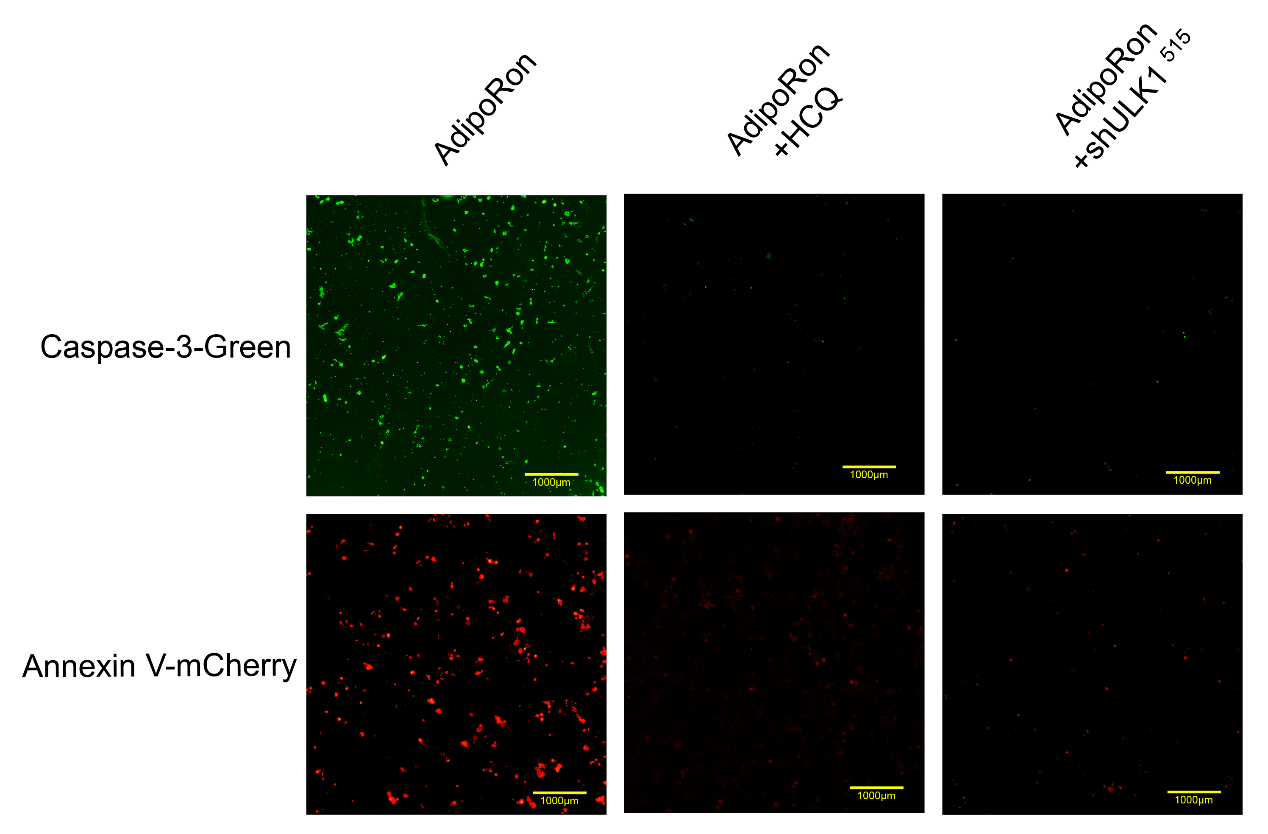


**Supplementary Figure 5. The regulatory role of AdipoRon on apoptosis-related proteins and autophagy in PTC cells.**

Caspase-3 activity and apoptosis in K-1 cells treated with AdipoRon for 40h (AdipoRon). AdipoRon +3MA K-1 cells were pretreated with 3MA (6 mM, 6 h). AdipoRon+HCQ K-1 cells were pretreated with HCQ (2 µM, 6 h). AdipoRon induced high Caspase-3 activity in the nuclei of apoptotic cells, showing bright green fluorescence, and Annexin V in the cell membrane of apoptotic cells, showing red fluorescence.
