# Supplementary material for: Uncovering the connection between obesity and thyroid cancer: the therapeutic potential of adiponectin receptor agonist in the AdipoR2-ULK axis
Source: Cell Death Dis. 2024 Sep 30;15(9):708. doi: 10.1038/s41419-024-07084-9 (PMC11443080; doi:10.1038/s41419-024-07084-9)
Supplement: Supplementary file 2 — Supplementary Tables [file 41419_2024_7084_MOESM2_ESM.docx]

**Supplementary Table 1. List of key resources used in this study**

| **REAGENTS or RESOURCES** | **SOURCE** | **IDENTIFIER** |
| --- | --- | --- |
| **Antibodies** | | |
| Anti-ASCT2 | CST | Cat#8057 |
| Anti-Atg12 | CST | Cat#4180 |
| Anti-Atg16L1 | CST | Cat#8089 |
| Anti-Atg3 | CST | Cat#3415 |
| Anti-Atg5 | CST | Cat#12994 |
| Anti-Glut1 | CST | Cat#12939 |
| Anti-GLS1 | CST | Cat#56750 |
| Anti-PKM2 | CST | Cat#4053 |
| Anti-LAT1 | CST | Cat#5347 |
| Anti-LDHA | CST | Cat#3582 |
| Anti-mtor | CST | Cat#2983 |
| Anti-p70 S6 Kinase | CST | Cat#4691 |
| Anti-Phospho-mtor (Ser2448) | CST | Cat#5536 |
| Anti-Phospho-p70 S6 Kinase (Thr389) | CST | Cat#9234 |
| Anti-Phospho-ULK1 (Ser555) | CST | Cat#5869 |
| Anti-Phospho-ULK1 (Thr180) | CST | Cat#88311 |
| Anti-Phospho-ULK1 (Ser317) | CST | Cat#89267 |
| Anti-Phospho-ULK1 (Ser467) | CST | Cat#4634 |
| Anti-Phospho-ULK1 (Ser638) | CST | Cat#14205 |
| Anti-Phospho-ULK1 (Ser757) | CST | Cat#6888S |
| Anti-SQSTM1/p62 | CST | Cat#8025 |
| Anti-ULK1 | CST | Cat#8054 |
| Anti- LC3A/B | CST | Cat#2772 |
| Anti-ADIPOR2 | ABCAM | Cat#ab77612 |
| Anti-ADIPOR1 | ABCAM | Cat#ab126611 |
| Anti-Beclin 1 | ABCAM | Cat#ab207612 |
| Anti- LC3B | NOVUS | Cat#NB100-2220 |
| Anti-igg (H+L), Alexa Fluor 488 | Invitrogen | Cat#A-11034 |
| Anti-BAX | PROTEINTECH | Cat#50599 |
| Anti-Bcl2 | PROTEINTECH | Cat#12789 |
| Anti-Caspase 3 | PROTEINTECH | Cat#66470 |
| Anti-Caspase 8 | PROTEINTECH | Cat#CL488-66093 |
| Anti- IGg | PROTEINTECH | Cat#30000-0-AP |
| **Chemicals, Peptides, and Recombinant Proteins** | | |
| Adiporon | ADIPOGEN | Cat#AG-CR1-0154-M050 |
| Chloroquine | Sigma-Aldrich | Cat#C6628 |
| 3-Methyladenine | Sigma-Aldrich | [Cat#5142-23-4](https://www.sigmaaldrich.cn/CN/zh/search/5142-23-4?focus=products&page=1&perpage=30&sort=relevance&term=5142-23-4&type=cas_number) |
| Lipofectamine 3000 | Invitrogen | [Cat#L3000001](https://www.thermofisher.cn/order/catalog/product/cn/zh/L3000001) |
| Alexa Fluor 647 Phalloidi | Invitrogen | Cat#A30107 |
| Dulbecco's Modified Eagle's Medium (DMEM) | GIBCO | Cat#C11995500BT |
| RPMI Medium 1640 | GIBCO | Cat#C11875500BT |
| TRYPSIN .5﹪ EDTA, 10X | GIBCO | Cat#15400-054 |
| Pageruler™ Prestained Protein Ladder, 10 to 180 kDa | Thermo Fisher Scientific | Cat#26616 |
| Pageruler™ Prestained Protein Ladder, 10 to 260 kDa | Thermo Fisher Scientific | Cat#26634 |
| RIPA Lysis and Extraction Buffer | Thermo Fisher Scientific | Cat#89901 |
| Dyed Red Aqueous Fluorescent Particles | Thermo Fisher Scientific | Cat#R100TS |
| SYBR™ Green Master Mix | Applied Biosystems | Cat#A25742 |
| Fugene® 6 Transfection Reagent | PROMEGA | Cat#E2691 |
| Rnaiso PLUS （TRZOL） | TAKARA | Cat#9109 |
| Lightcycler 8-Tube Strips (white) | Roche | Cat#6612601001 |
| General Antibody Dilution Buffer | NCM | Cat#WBS00D |
| Certified Foetal Bovine Serum | BI | Cat#04-001-1A |
| Western chemiluminescent HRP substrate kit (ECL) | Millipore | Cat#P90719 |
| Phosphatase Inhibitor Cocktail (2 Tubes, 100X) | APEXBIO | Cat#K1012 |
| Protease Inhibitor Cocktail (EDTA-Free,100X in DMSO) | APEXBIO | Cat#K1007 |
| Triton X-100 | Solarbio | Cat#T8022 |
| DAPI Staining Solution | Solarbio | Cat#C0065 |
| Paraformaldehyde | Solarbio | Cat#P1110 |
| **Critical Commercial Assays** | | |
| Genejet RNA Purification Kit | Thermo Fisher Scientific | Cat#k0731 |
| Reverted First-Strand cDNA Synthesis Kit | Thermo Fisher Scientific | Cat#K1622 |
| BCA Protein Assay Kit | Thermo Fisher Scientific | Cat#23227 |
| Cell Counting Kit-8 | Dojindo | Cat#CK04 |
| RNA 6000 Nano Lab Chip Kit | Agilent | Cat#5067-1511 |
| Nebnext Ultra II RNA Library Prep Kit | NEB | Cat#E7760 |
| Plasmid Extraction Kit | TIANGEN | Cat#DP112 |
| G6P Assay Kit | Beyotime | Cat#S0185 |
| NAD^+^/NADH Assay Kit | Beyotime | Cat#S0175 |
| Caspase-3 Activity and Apoptosis Detection Kit | Beyotime | Cat#C1077S |
| Glucose Assay Kit | Beyotime | Cat#S0201S |
| Edu Cell Proliferation Kit | Invitrogen | Cat#C10337 |
| Fluovolt™ Membrane Potential Kit | Invitrogen | Cat#F10488 |
| **Experimental Models: Organisms/Strains** | | |
| Escherichia coli strain DH5a | TIANGEN | Cat#CB101 |
| Sequences of siRNAs, see Table S1 | Genepharma | N/A |
| Sequences of shRNAs, see Table S1 | AMOGENE | N/A |
| Sequences of sgRNAs, see Table S1 | AMOGENE | N/A |
| Sequences of PCR primer, see Table S1 | Sangon | N/A |
| **Software and Algorithms** | | |
| Image J | National Institutes of Health | Https://imagej.nih.gov/ij/ |
| R | R Development Core Team | Https://www.r-project.org/ |
| Microsoft Excel | Microsoft | [Https://www.microsoft.com/](https://www.microsoft.com/) |
| Graphpad Prism | Graphpad Software | Https://www.graphpad.com/ |
| SPSS | [IBM](https://baike.baidu.com/item/IBM/9190) | [Https://www.ibm.com/](https://www.ibm.com/) |

**Supplementary Table 2. The Quantibody® Human Obesity Array 3**

| **NO.** | **Contents** |
| --- | --- |
| 1 | Quantibody^®^Array Glass Chip |
| 2 | Sample Diluent |
| 3 | 20X Wash Buffer I |
| 4 | 20X Wash Buffer II |
| 5 | Lyophilized cytokine standard mix * |
| 6 | Detection antibody cocktail |
| 7 | Cy3equivalent dye-conjugated Streptavidin |
| 8 | Slide washer/Dryer |
| 9 | Adhesive device sealer |

Supplementary Table 3. Forty kinds of adipokines in adipokine antibody array.

| Adipokine abbreviation | Another name for adipokine |
| --- | --- |
| Adiponectin,AdipoQ | ADIPOQ ACDC ACRP30 APM1 GBP28 |
| Adipsin | CFD DF PFD |
| AgRP | AGRP AGRT ART |
| ANGPTL4 | ANGPTL4 ARP4 HFARP PGAR PP1158 PSEC0166 UNQ171/PRO197 |
| BDNF | BDNF |
| Chemerin | RARRES2 TIG2 |
| CRP | CRP PTX1 |
| GH | GH1 |
| IFNg | IFNG |
| IGFBP-1 | IGFBP1 IBP1 |
| IGFBP-2 | IGFBP2 BP2 IBP2 |
| IGF-1 | IGF1 IBP1 |
| IL-10 | IL10 |
| IL-12p40 | IL12B NKSF2 |
| IL-12p70 | IL12A/IL12B |
| IL-1b | IL1B IL1F2 |
| IL-1ra | IL1RN IL1F3 IL1RA |
| IL-6 | IL6 IFNB2 |
| IL-8 | CXCL8 IL8 |
| Insulin | INS |
| Leptin | LEP OB OBS |
| Lipocalin-2 | LCN2 HNL NGAL |
| MSP | MST1 D3F15S2 DNF15S2 HGFL |
| OPG | TNFRSF11B OCIF OPG |
| PAI-1 | SERPINE1 PAI1 PLANH1 |
| PDGF-BB | PDGFB PDGF2 SIS |
| Pepsinogen I | PGA4/PGA3/PGA5 |
| Pepsinogen II | PGC |
| Procalcitonin | CALCA CALC1 |
| Prolactin | PRL |
| RANTES | CCL5 D17S136E SCYA5 |
| RBP4 | RBP4 PRO2222 |
| Resistin | RETN FIZZ3 HXCP1 RSTN UNQ407/PRO1199 |
| SAA | SAA1 |
| TGFb1 | TGFB1 TGFB |
| TSP-1 | THBS1 TSP TSP1 |
| TNF RI | TNFRSF1A TNFAR TNFR1 |
| TNF RII | TNFRSF1B TNFBR TNFR2 |
| TNFa | TNF TNFA TNFSF2 |
| VEGF | VEGFA VEGF |

**Supplementary Table 4. The sequences of qPCR primers and shRNA**

| **No.** | **Gene name** | **Sequence** | |
| --- | --- | --- | --- |
| qPCR Primers | | Sense（5'-3'） | Antisense（5'-3'） |
| 1 | ADIPOR2 | CCCTCATGATGTACTACCAGAC | GTTGCCTGTTTCTGTGTGTATT |
| 2 | ADIPOR1 | AAGCTGAAGAAGAGCAAACATG | ATGACCATGTAGCAGATAGTCG |
| 3 | GLUT-1 | TGTCTGGCATCAACGCTGTCTTC | CCTGCTCGCTCCACCACAAAC |
| 4 | PKM2 | ACTGGCATCATCTGTACCATTG | AGCCACATTCATTCCAGACTTA |
| 5 | LDHA | AGGTGATCAAACTCAAAGGCTA | CCCAAAATGCAAGGAACACTAA |
| 6 | PDK1 | AACCGACACAATGATGTCATTC | ATGCGACTCATGTAGAATCGAT |
| 7 | GLS | CACTCAAATCTACAGGATTGCG | CCAGACTGCTTTTTAGCACTTT |
| 8 | SLC1A5 | CAGTCCTTGGACTTCGTAAAGA | CCAGGATCAAGGAGATATGGTC |
| 9 | SLC7A5 | ATGATCAACCCCTACAGAAACC | CAGGTGATAGTTCCCGAAGTC |
| 10 | Tg | AAGGGCGGGAACTGGCTGAG | TGACTGATTGAACTGCGAGGAACC |
| 11 | TPO | ACCGCTATTCTGACCTCCTGATGG | TTTGCTGGTGCTCTGTGGTGTG |
| 12 | TSHR | GAGTTATCGGTGTATACGCTGA | ACTGACTTTGGCATAGCTACTT |
| 13 | ULK1 | CTCCTTTGACTTCCCGAAGAC | CTTAAGGAGCAGGTCAGTGAG |
| shRNA | | Sense（5'-3'） | Antisense（5'-3'） |
| 1 | ULK1-Homo-1884 | UACACGCCAUCUCCUCAAGUUTT | AACUUGAGGAGAUGGCGUGUATT |
| 2 | ULK1-Homo-515 | CAAGUGCAUUAACAAGAAGAATT | UUCUUCUUGUUAAUGCACUUGTT |
| 3 | ULK1-Homo-3191 | CAGGCUGAAUGAGCUGUACAATT | UUGUACAGCUCAUUCAGCCUGTT |
| 4 | ADIPOR1-Homo-591 | CAUAUGAUGUGCUCCCUGATT | UCAGGGAGCACAUCAUAUGTT |
| 5 | ADIPOR1-Homo-773 | CUCAGACCAAAUAUGUACUTT | AGUACAUAUUUGGUCUGAGTT |
| 6 | ADIPOR1-Homo-968 | CCCUGGCUCUAUUAUUCCUTT | AGGAAUAAUAGAGCCAGGGTT |
| 7 | ADIPOR2-Homo-280 | GGUACACGAAGAGGUGAUATT | UAUCACCUCUUCGUGUACCTT |
| 8 | ADIPOR2-Homo-647 | CACAUCUCUUAGGUUGUGUTT | ACACAACCUAAGAGAUGUGTT |
| 9 | ADIPOR2-Homo-971 | CAGCCAUUAUAGUCUCCCATT | UGGGAGACUAUAAUGGCUGTT |

**Supplementary Table 5. Adipokine antibody array detection sequence**

| Group | NO. | Group | NO. | Group | NO. | Group | NO. |
| --- | --- | --- | --- | --- | --- | --- | --- |
| *Male tissue normal group*  *(N=7)* | 100234359 | *Male tissue obesity group*  ***(N=7)*** | 100245966 | *Female tissue normal grou****p***  ***(N=7)*** | 100230619 | *Female tissue obesity group*  ***(N=7)*** | 100239772 |
|  | 100206935 |  | 100252251 |  | 100121369 |  | 100238911 |
|  | 100239722 |  | 100145533 |  | 100108072 |  | 100238477 |
|  | 100251058 |  | 100185120 |  | 100017929 |  | 100238431 |
|  | 100251058 |  | 100250783 |  | 100236814 |  | 100244488 |
|  | 100234359 |  | 100111771 |  | 100236693 |  | 100235982 |
|  | 100206935 |  | 100254499 |  | 100231696 |  | 100256142 |
