# Supplementary material for: Uncovering the connection between obesity and thyroid cancer: the therapeutic potential of adiponectin receptor agonist in the AdipoR2-ULK axis
Source: Cell Death Dis. 2024 Sep 30;15(9):708. doi: 10.1038/s41419-024-07084-9 (PMC11443080; doi:10.1038/s41419-024-07084-9)

Raw images of the immunoblotting experiments

Figure 4G

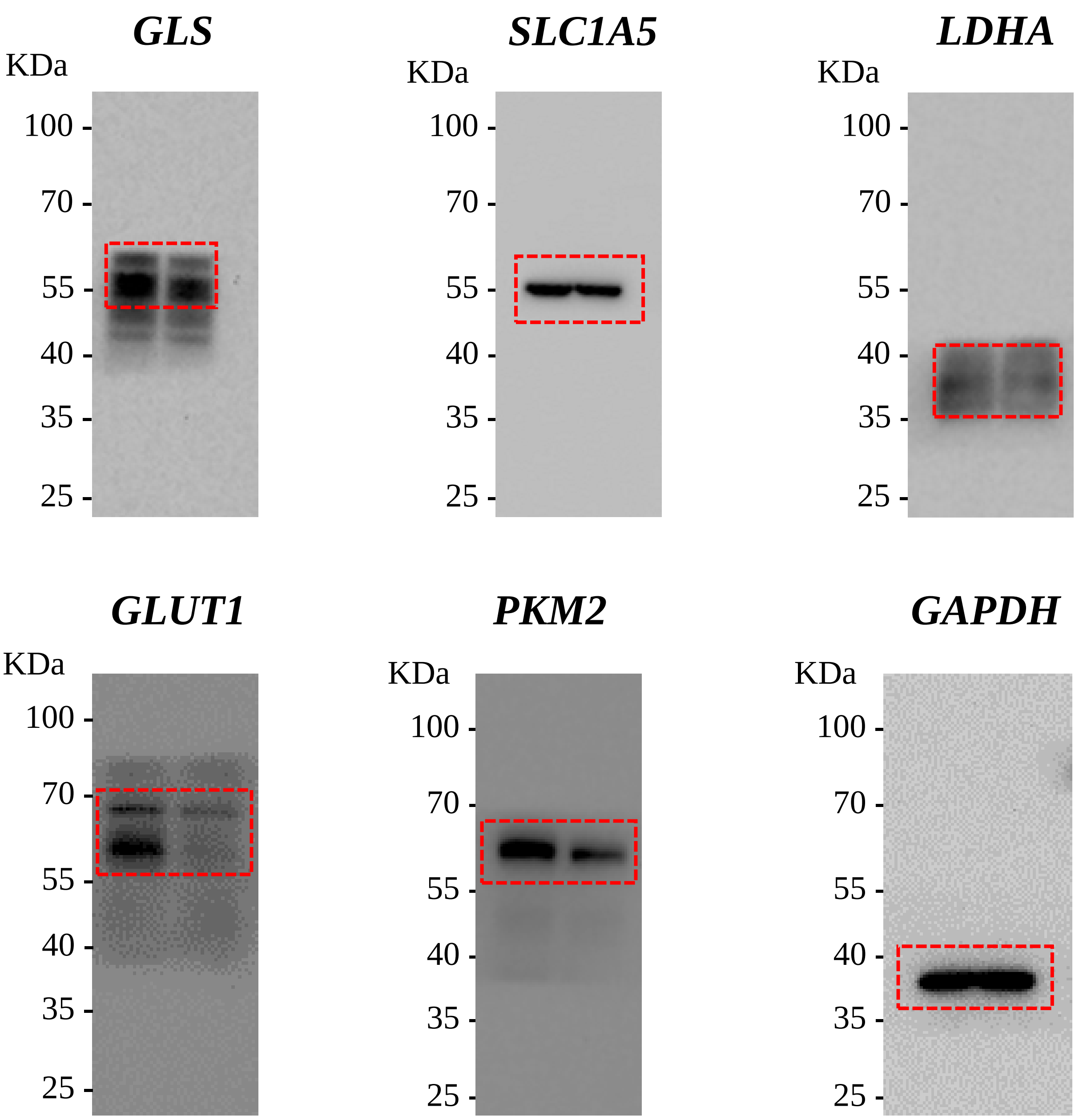

Figure 5G

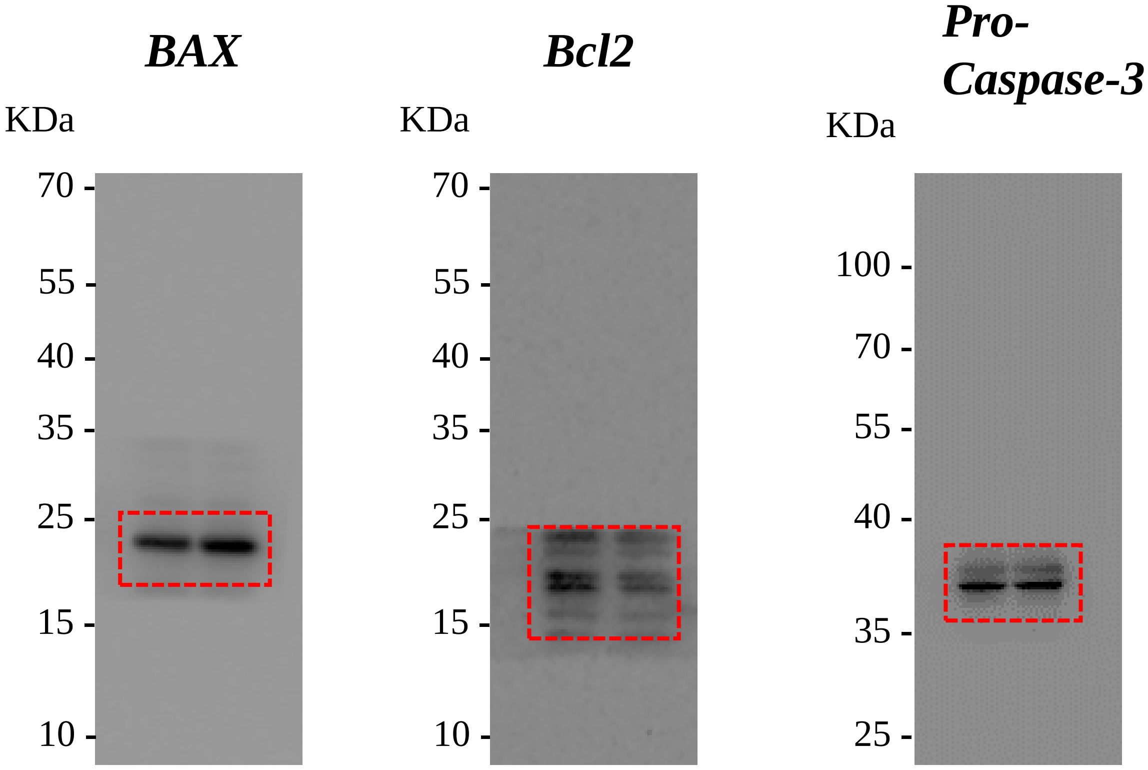

Figure 5G

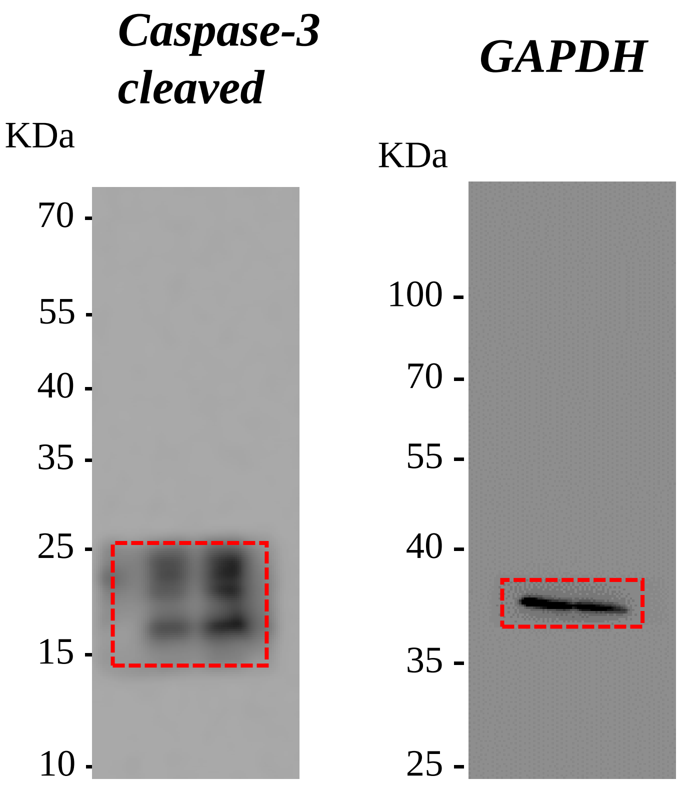

Figure 7A

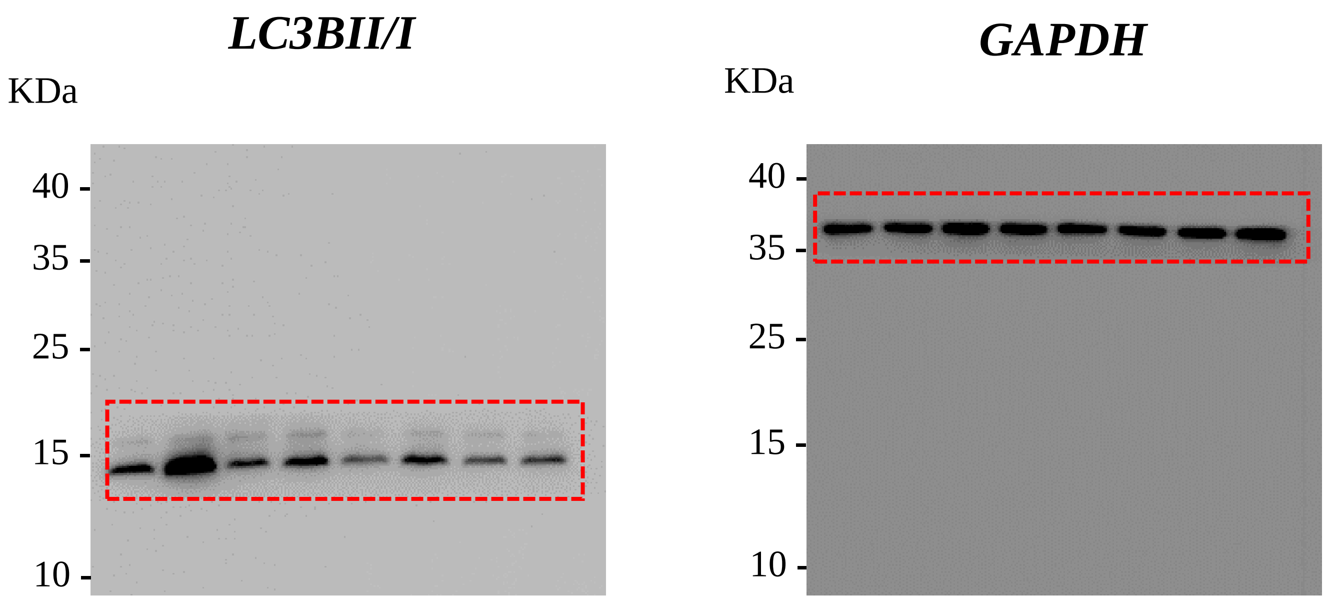

Figure 7C

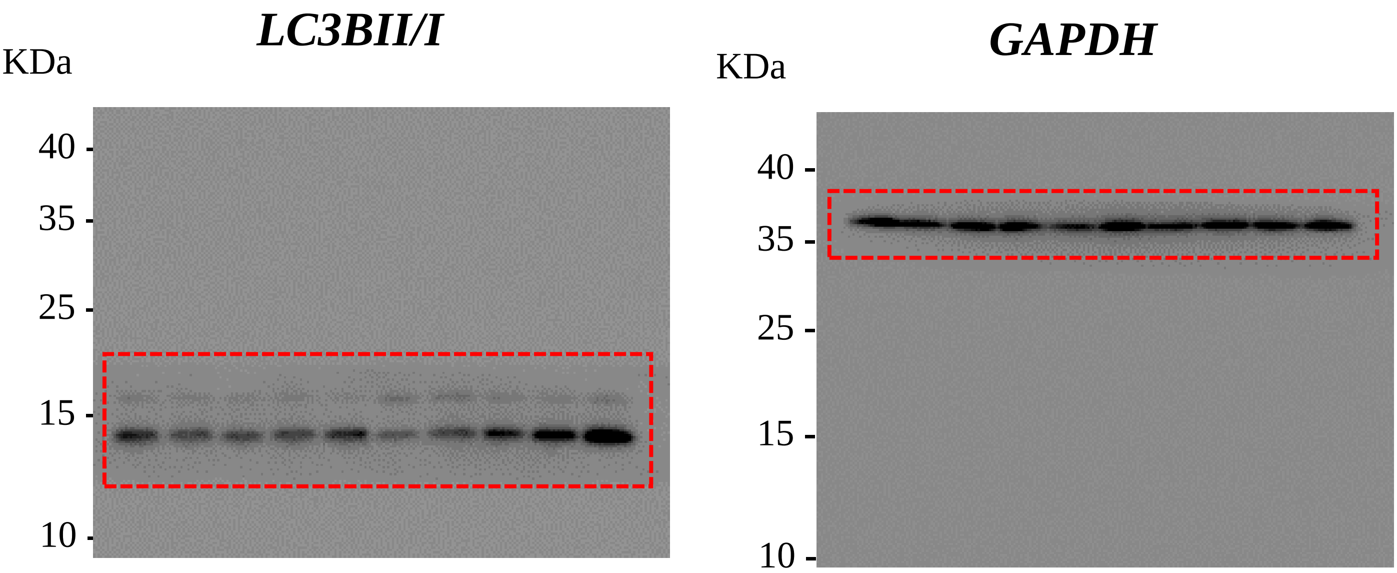

Figure 7D

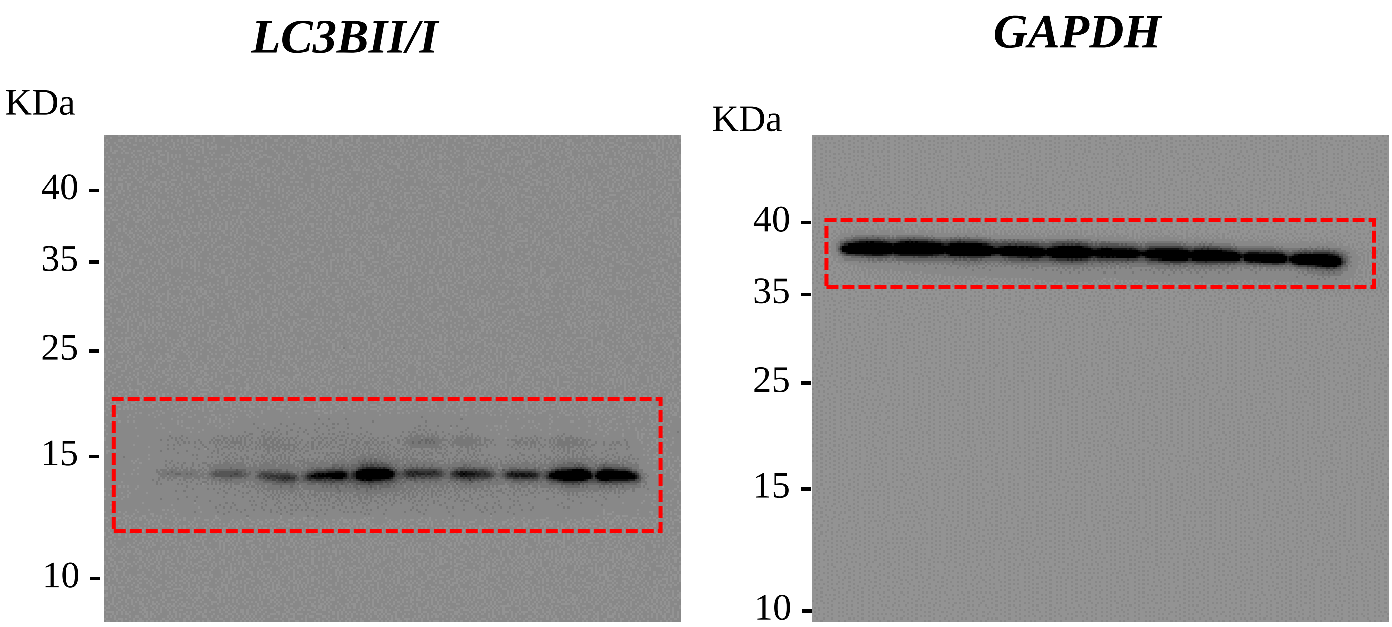

Figure 7F

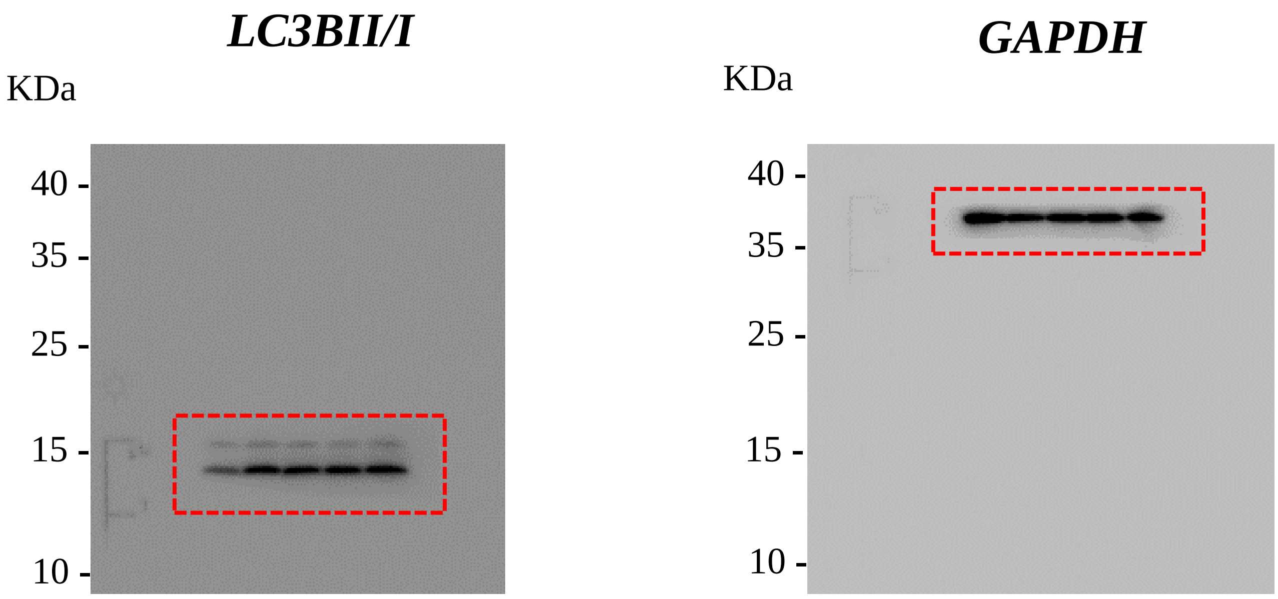

Figure 7G

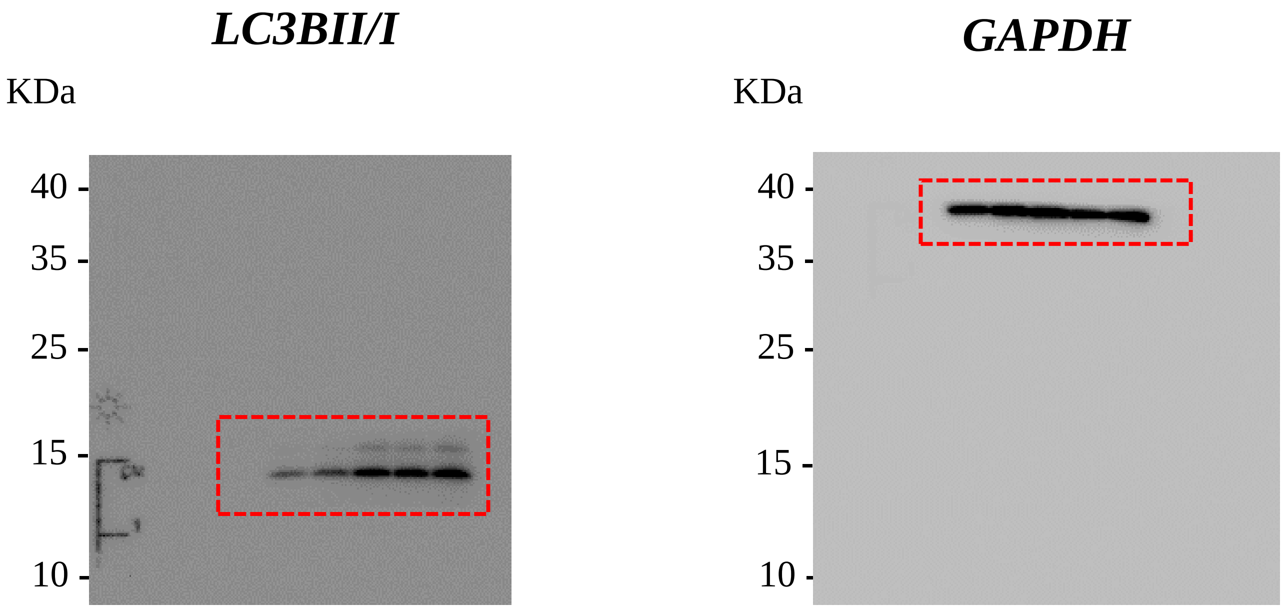

Figure 8C

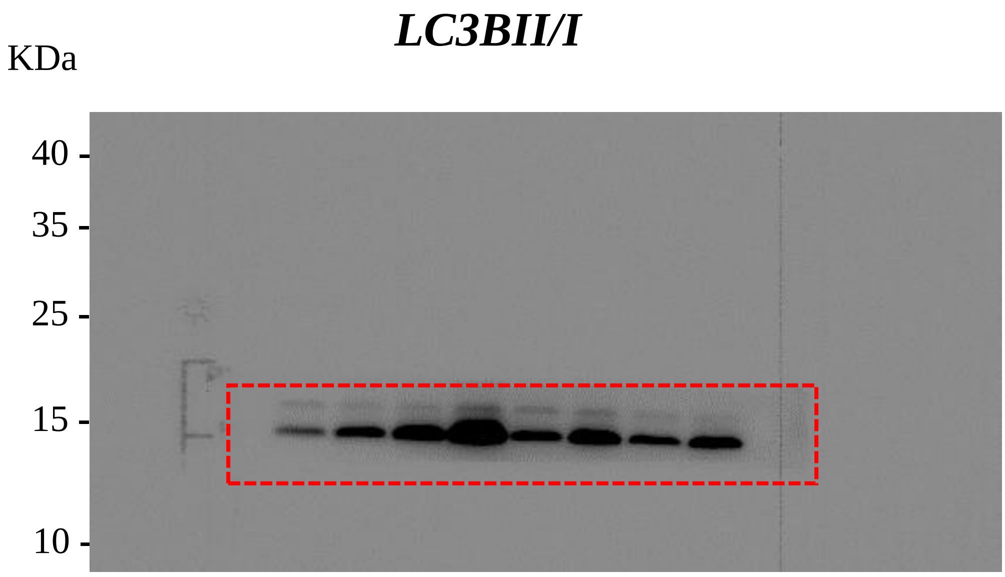

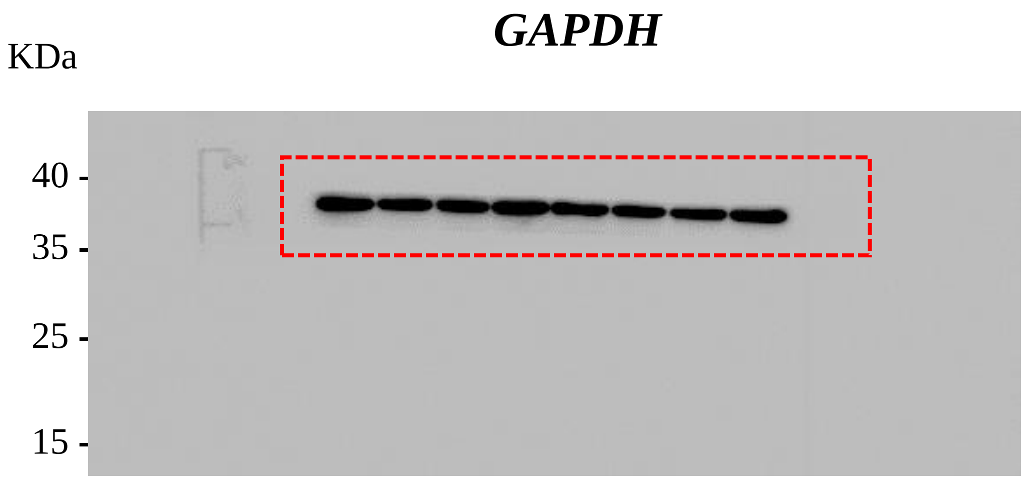

Figure 8D

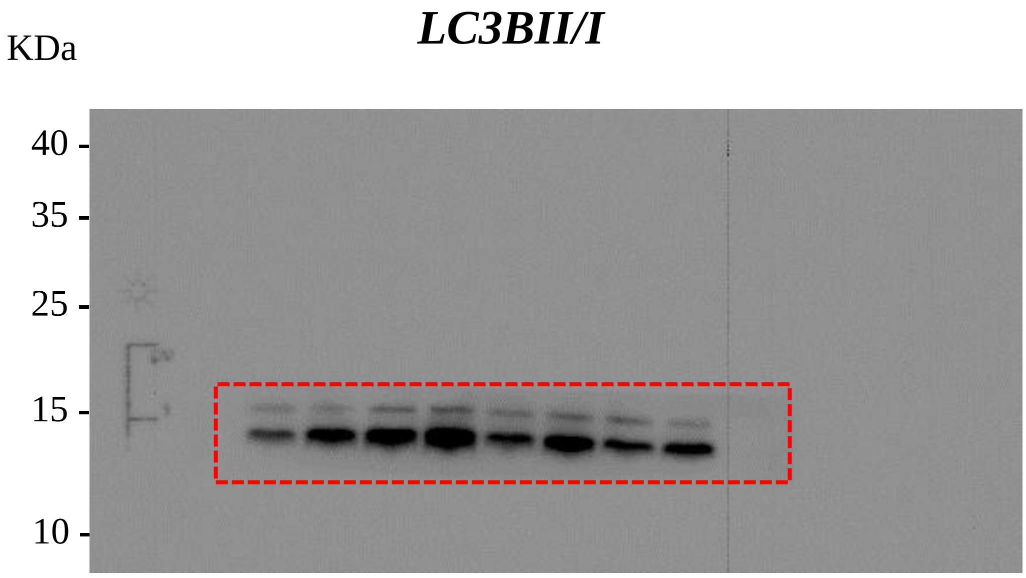

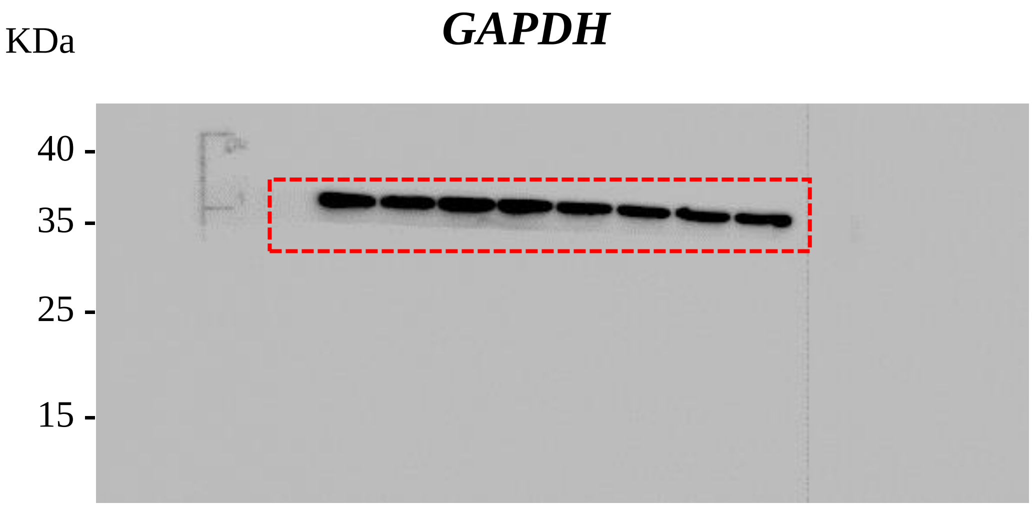

Figure 9A

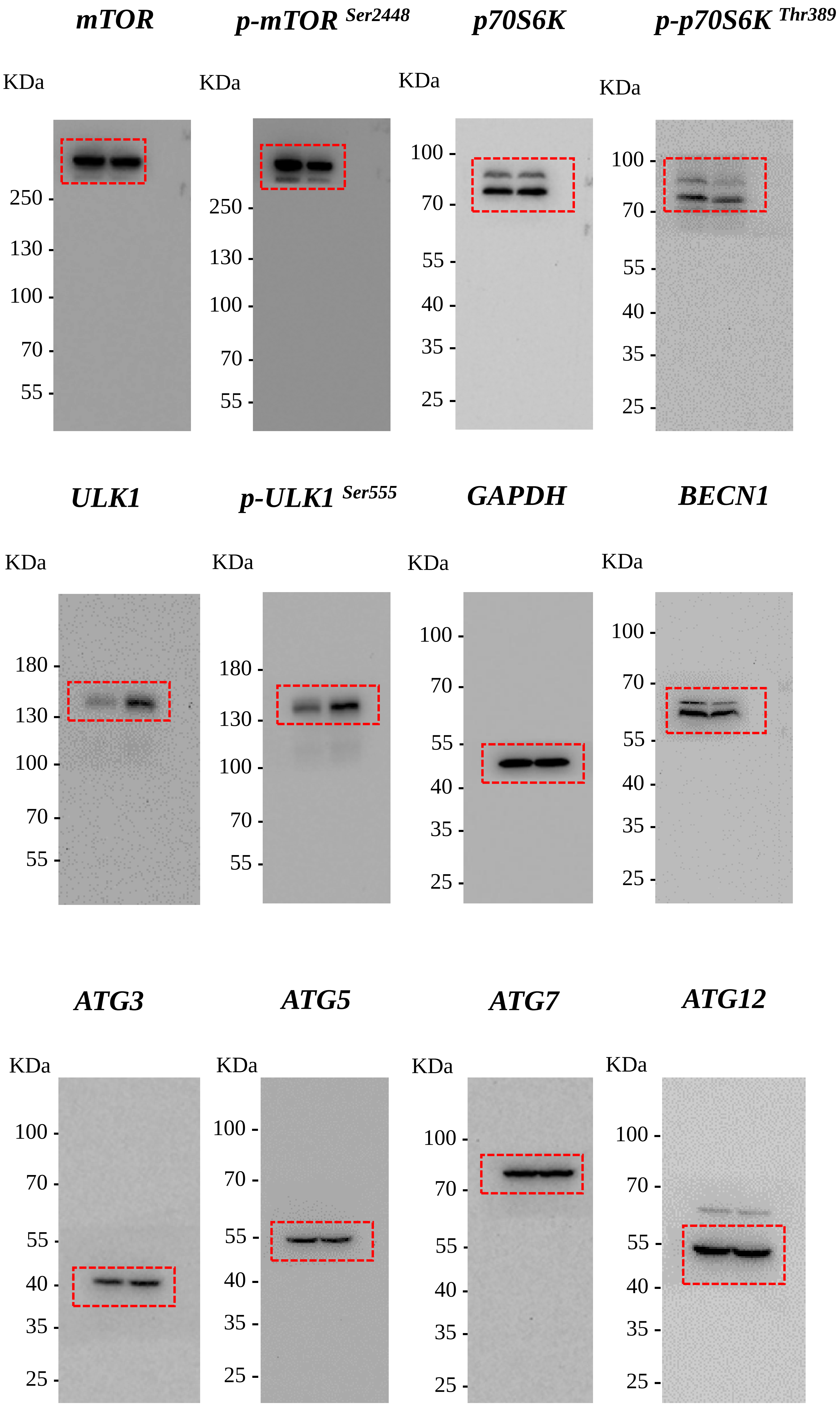
mTOR
p-mTOR Ser2448
p70S6K
p-p70S6K Thr389
KDa
250
130
100
70
55
KDa
250
130
100
70
55
KDa
100
70
55
40
35
25
KDa
100
70
55
40
35
25
ULK1
p-ULK1 Ser555
GAPDH
BECN1
KDa
180
130
100
70
55
KDa
180
130
100
70
55
KDa
100
70
55
40
35
25
KDa
100
70
55
40
35
25
ATG3
ATG5
ATG7
ATG12
KDa
100
70
55
40
35
25
KDa
100
70
55
40
35
25
KDa
100
70
55
40
35
25
KDa
100
70
55
40
35
25

Figure 9A

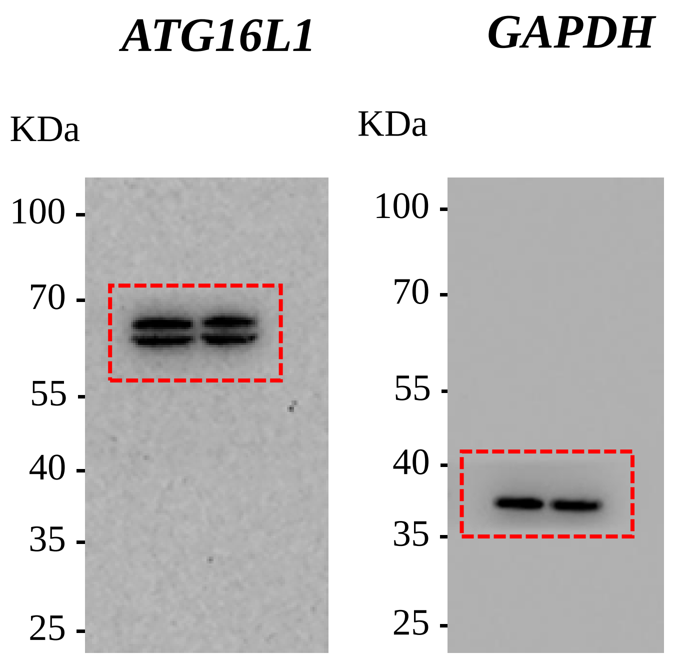

Figure 9B

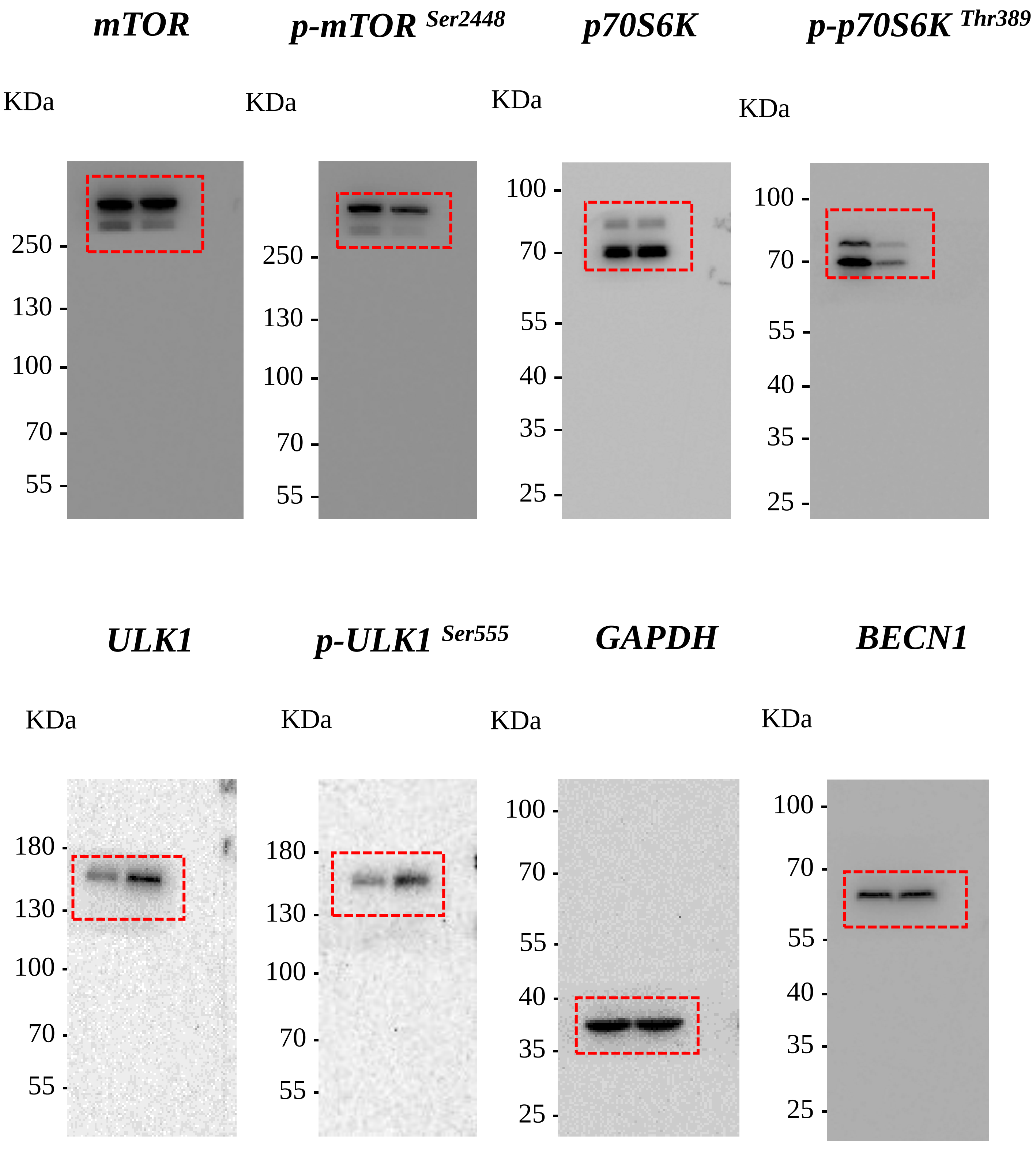

Figure 9B

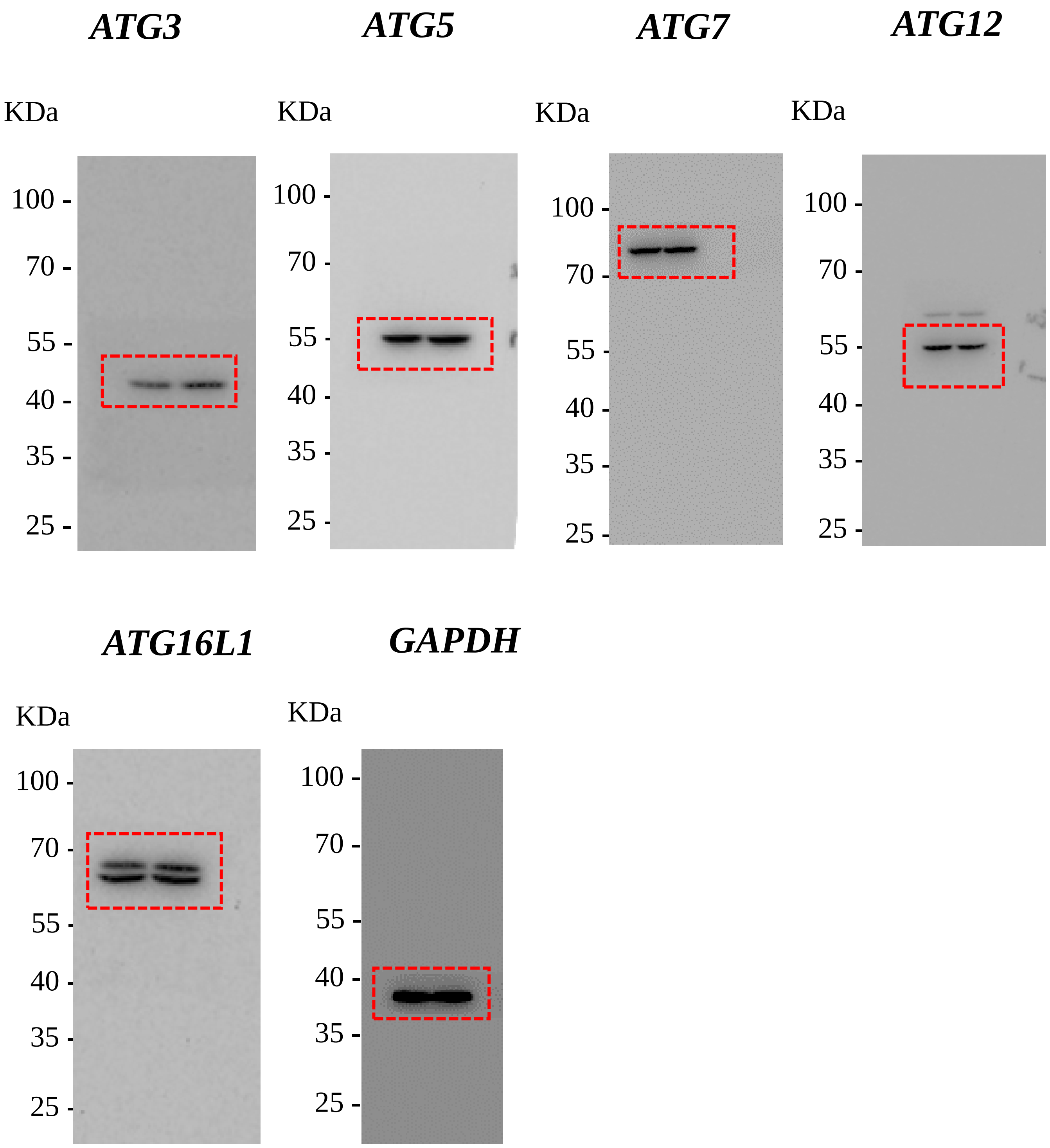

Figure 9C

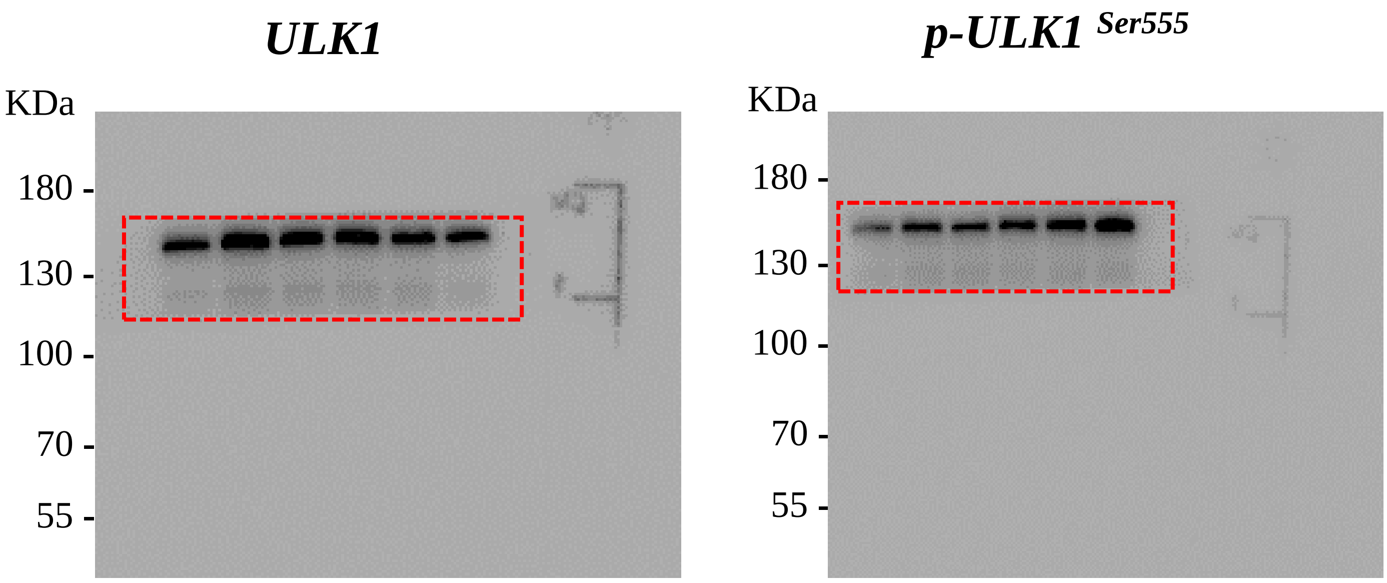

Figure 9C

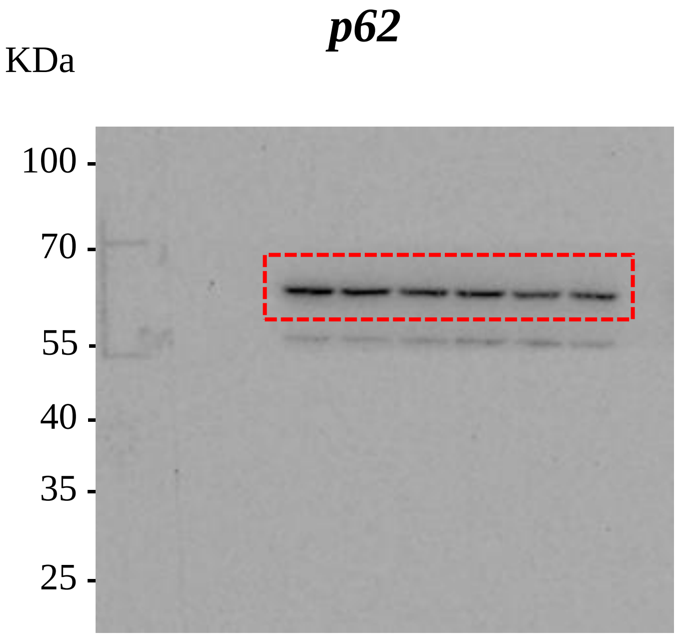

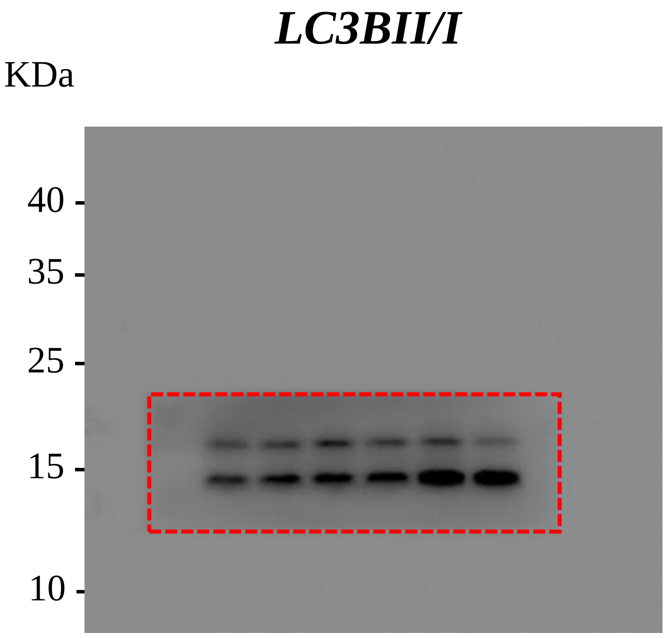

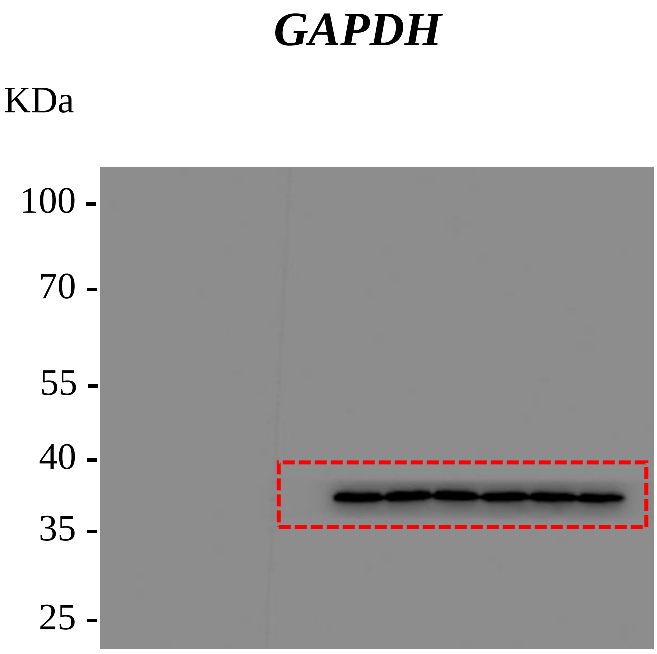

Figure 10A

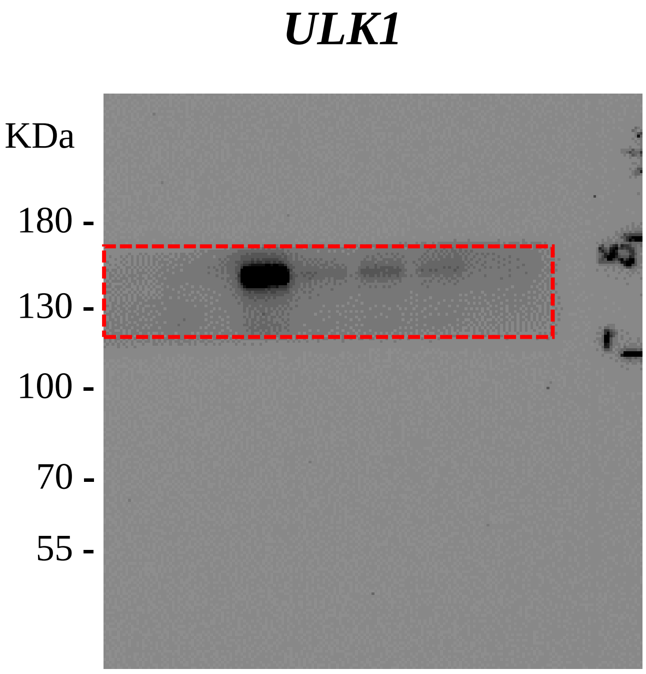

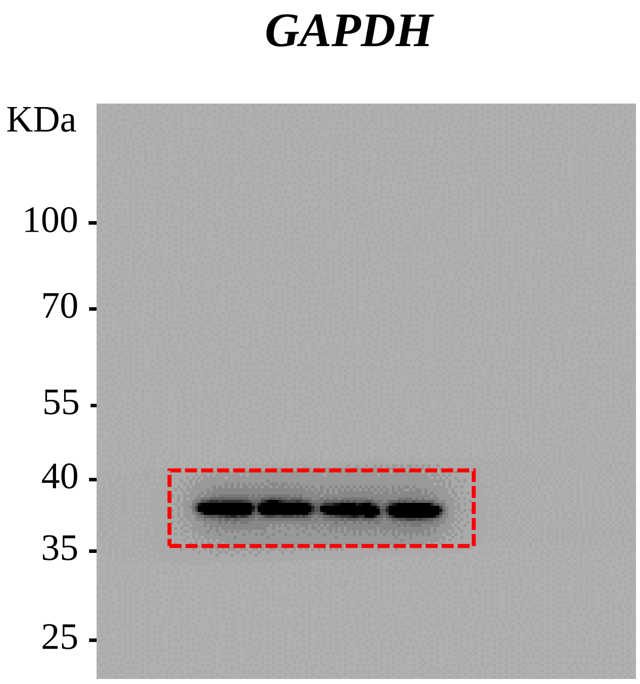

Figure 10C

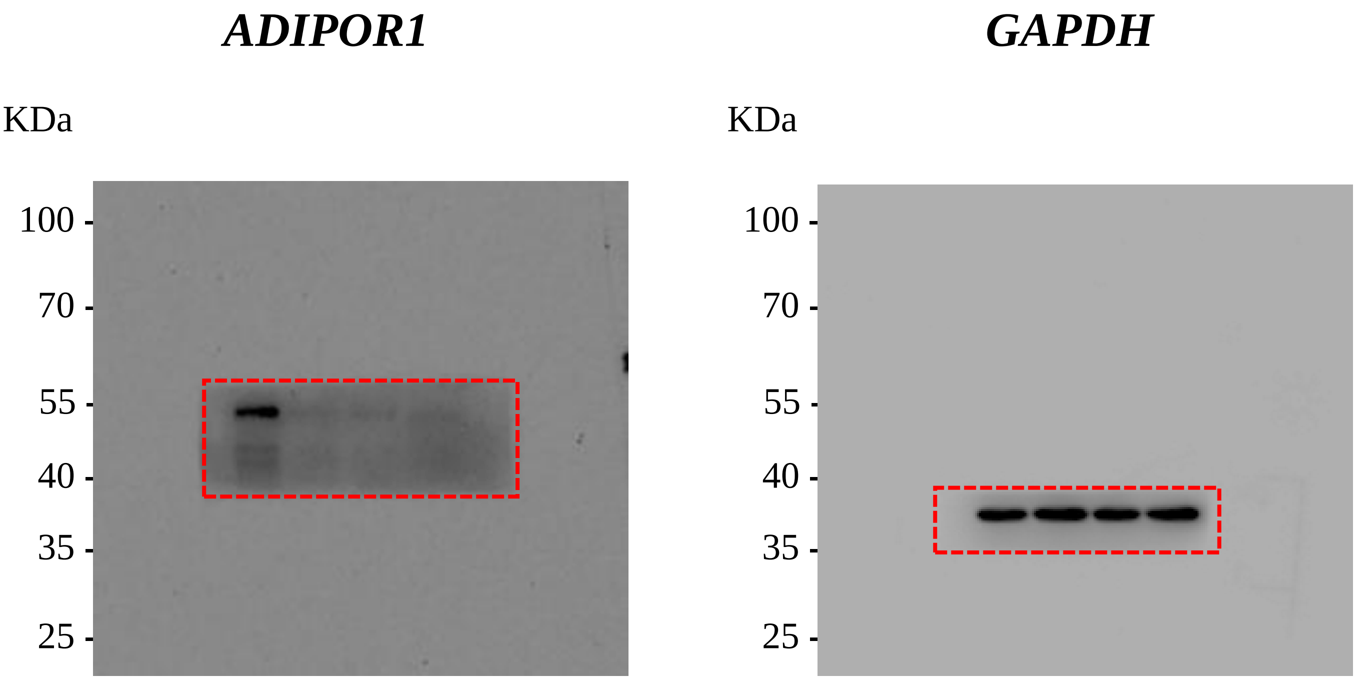

Figure 10E

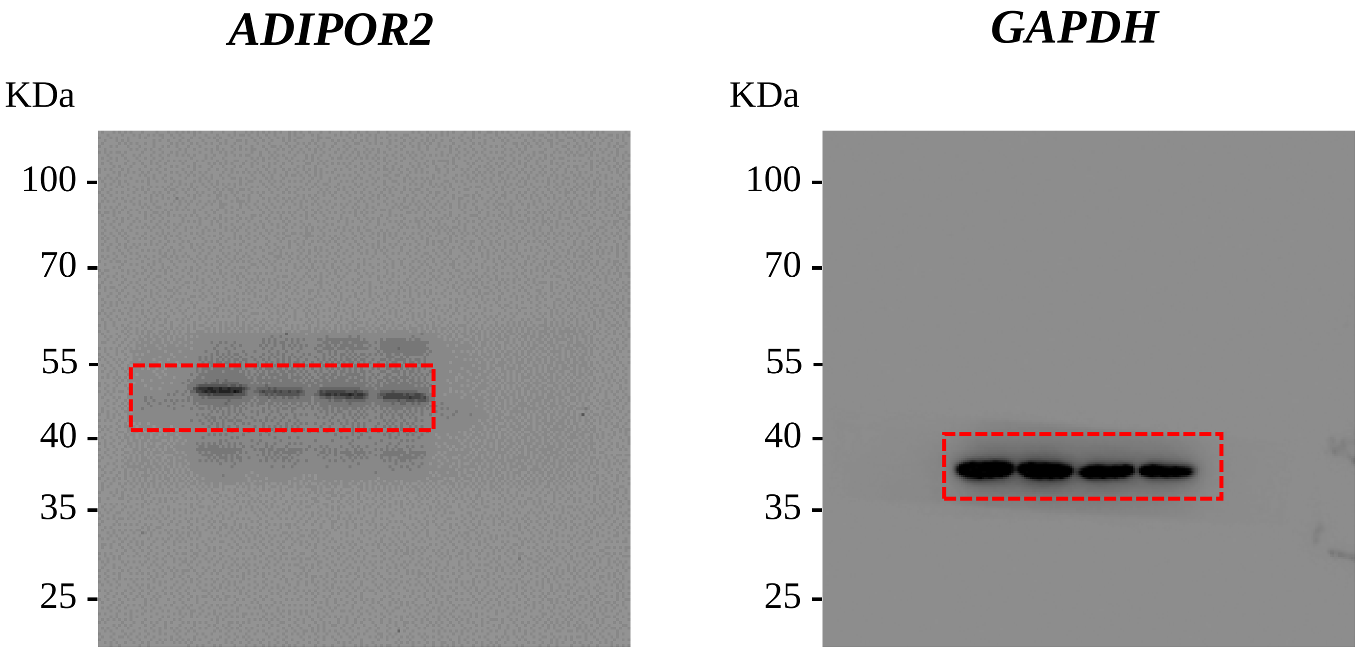

Figure 10G

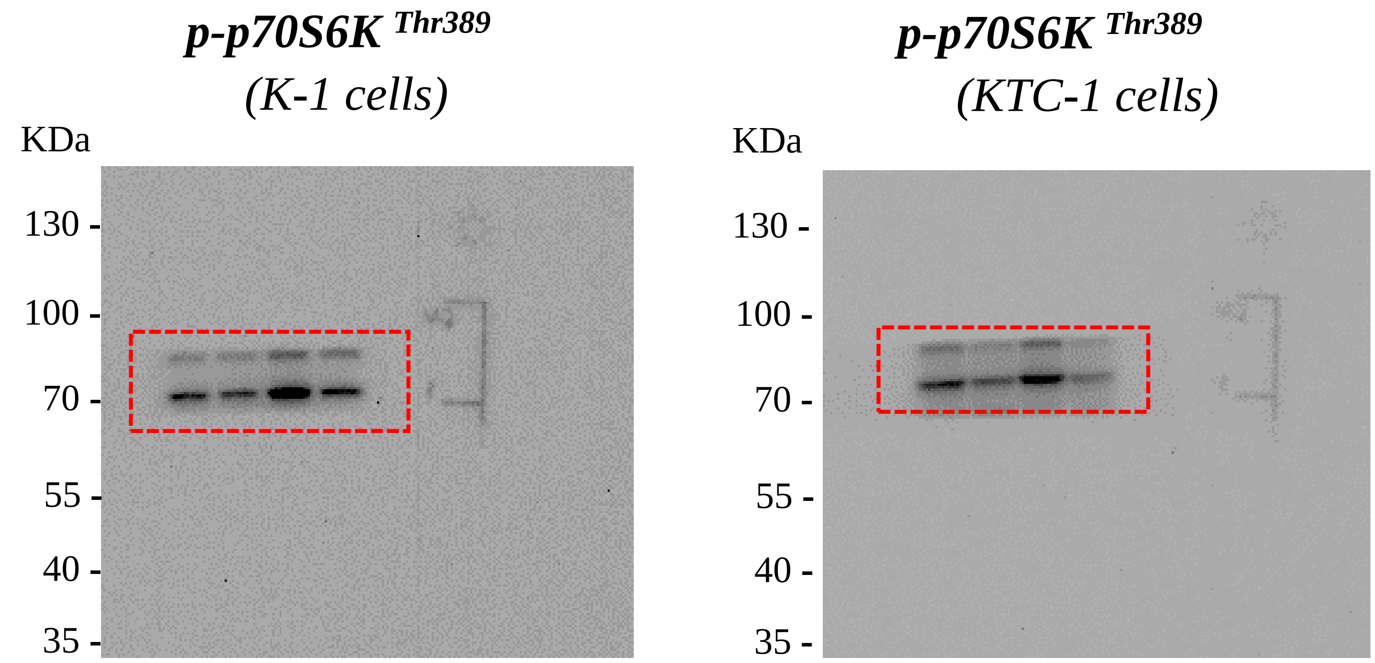

Figure 10G

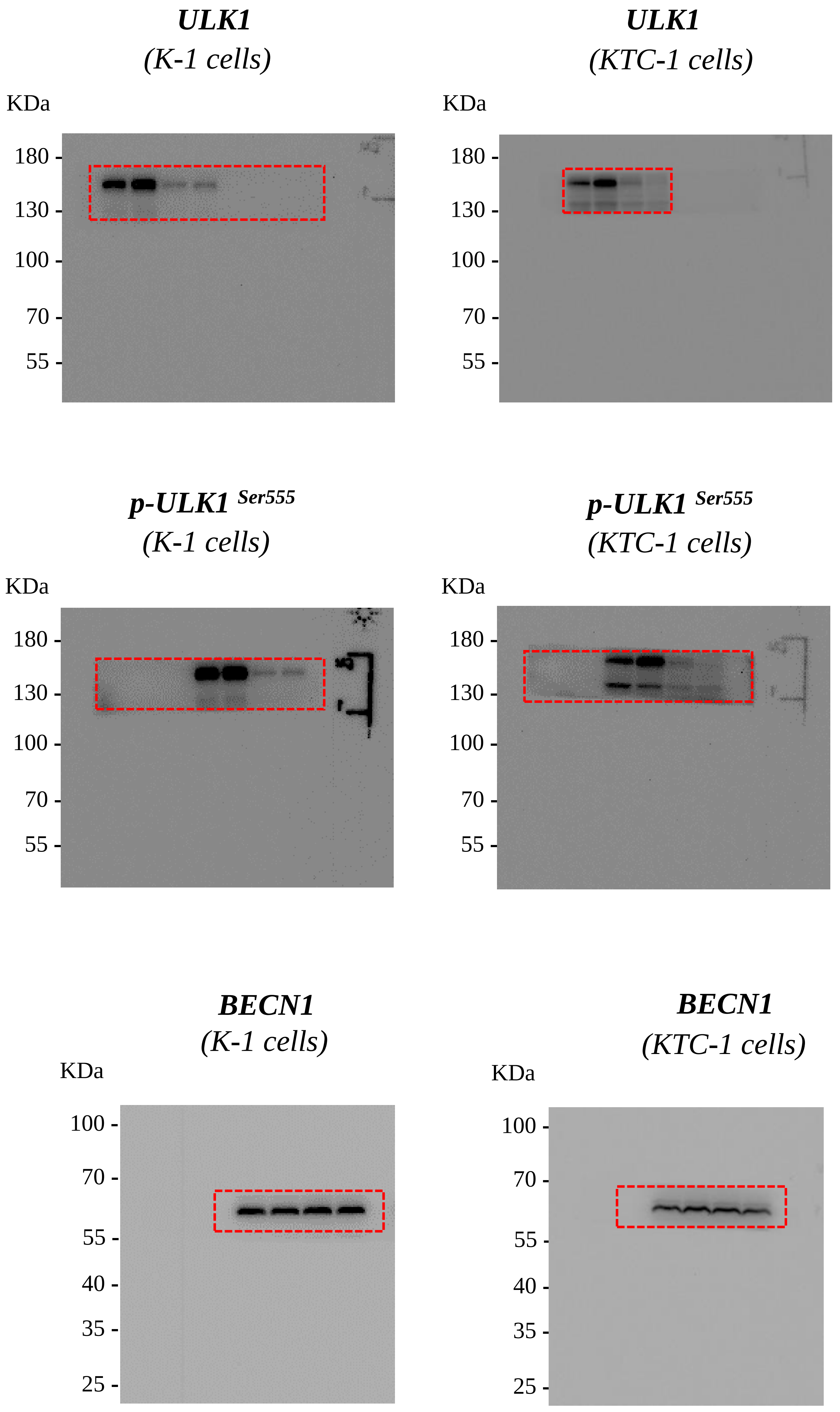

ULK1
(K-1 cells)
KDa
180
130
100
70
55
ULK1
(KTC-1 cells)
KDa
180
130
100
70
55
p-ULK1 Ser555
(K-1 cells)
KDa
180
130
100
70
55
p-ULK1 Ser555
(KTC-1 cells)
KDa
180
130
100
70
55
BECN1
(K-1 cells)
KDa
100
70
55
40
35
25
BECN1
(KTC-1 cells)
KDa
100
70
55
40
35
25

Figure 10G

LC3BII/I
(K-1 cells)

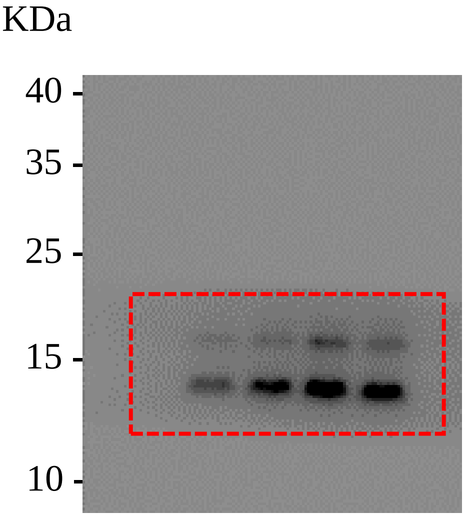

LC3BII/I
(KTC-1 cells)

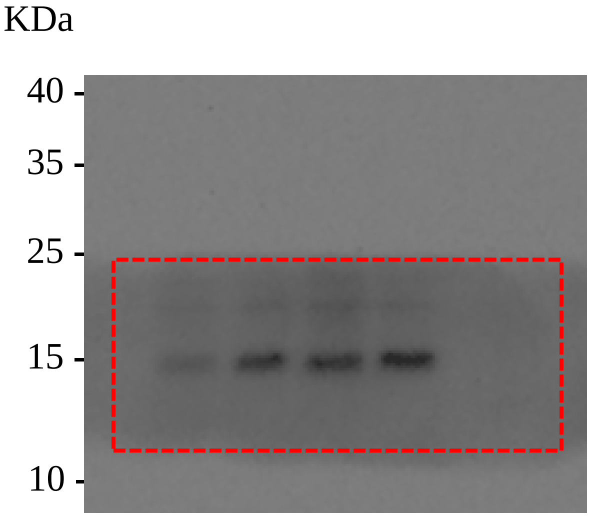

GAPDH
(K-1 cells)

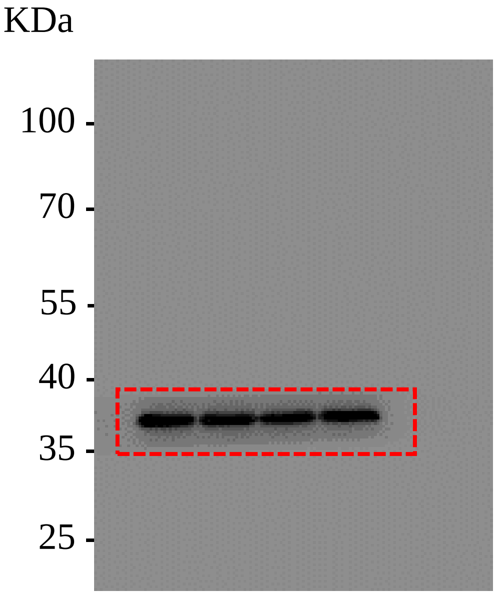

GAPDH
(KTC-1 cells)

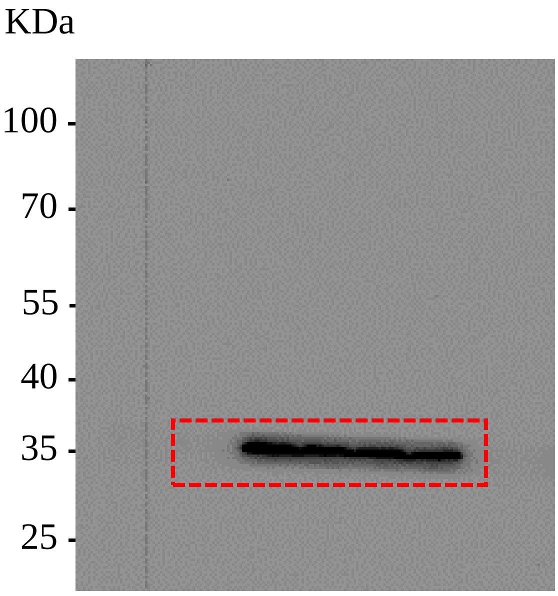

Figure 10I

ULK1
(K-1 cells)

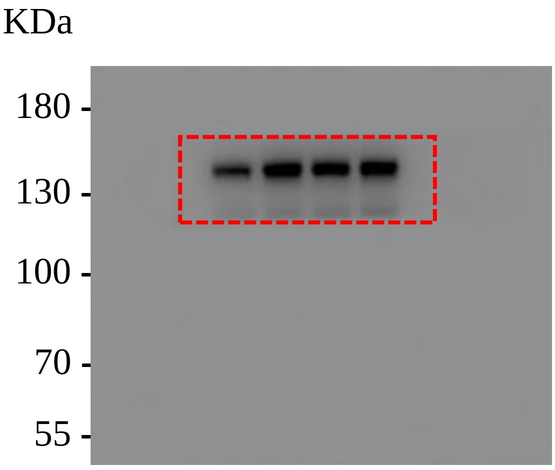

ULK1
(KTC-1 cells)

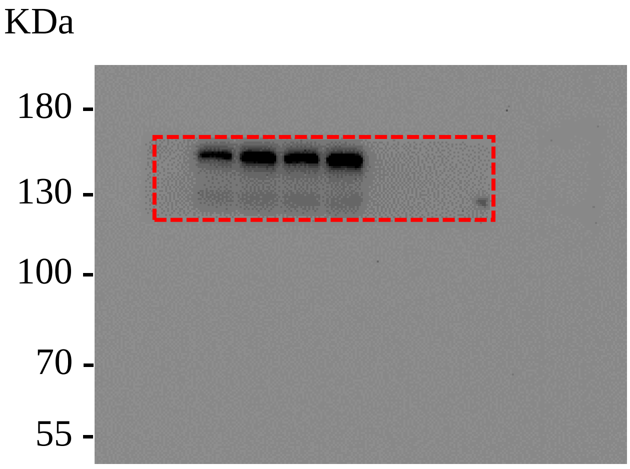

Figure 10I

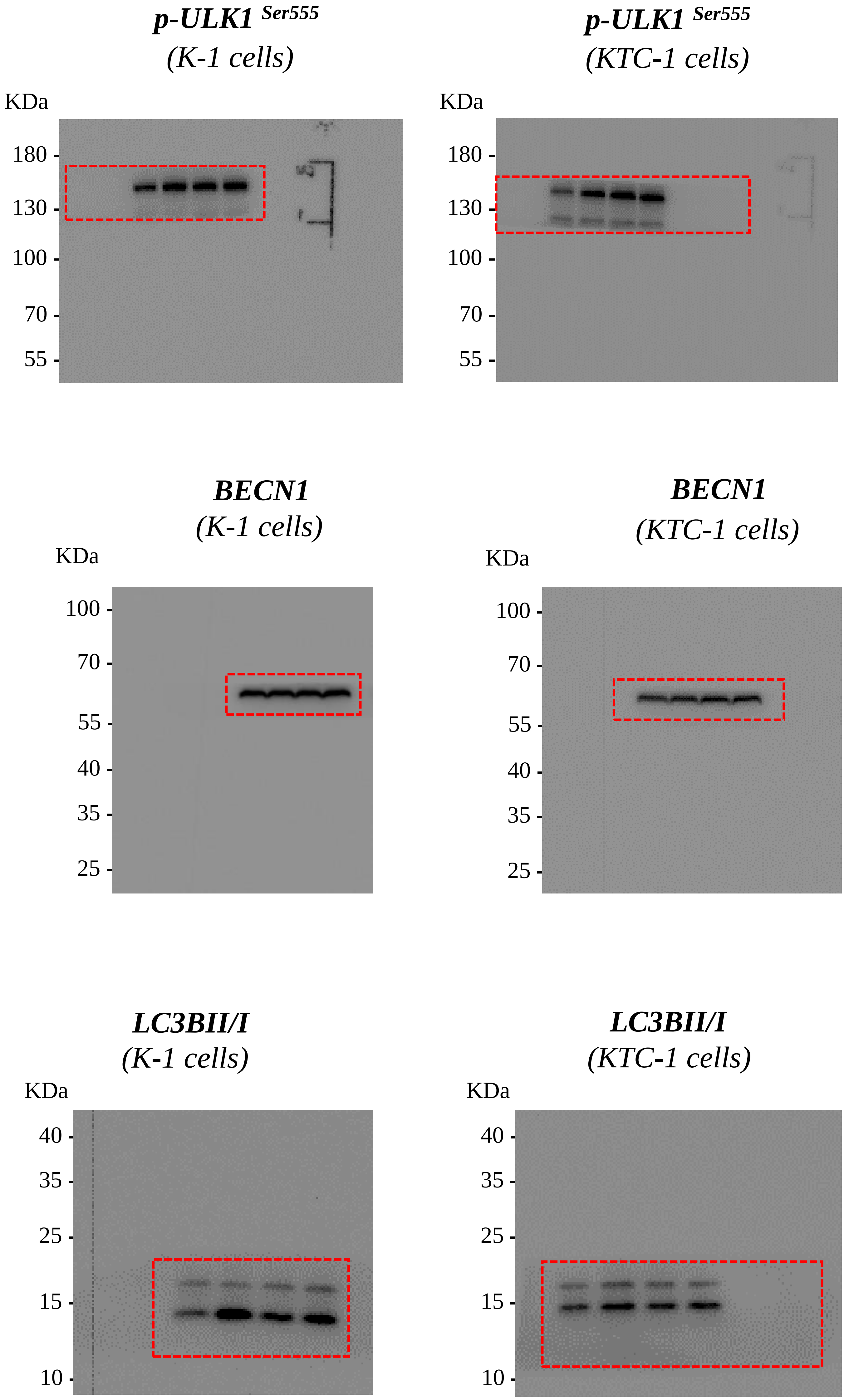
p-ULK1 Ser555
(K-1 cells)
KDa
180
130
100
70
55
p-ULK1 Ser555
(KTC-1 cells)
KDa
180
130
100
70
55
BECN1
(K-1 cells)
KDa
100
70
55
40
35
25
BECN1
(KTC-1 cells)
KDa
100
70
55
40
35
25
LC3BII/I
(K-1 cells)
KDa
40
35
25
15
10
LC3BII/I
(KTC-1 cells)
KDa
40
35
25
15
10

Figure 10I

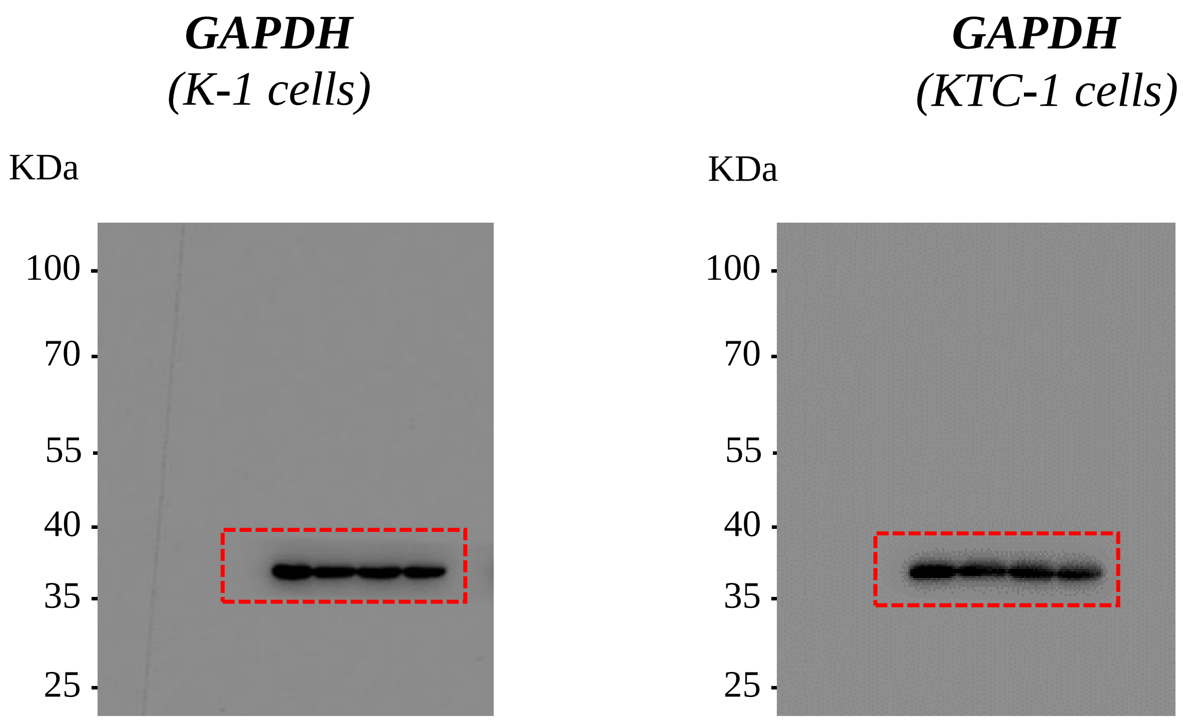

Figure 11A

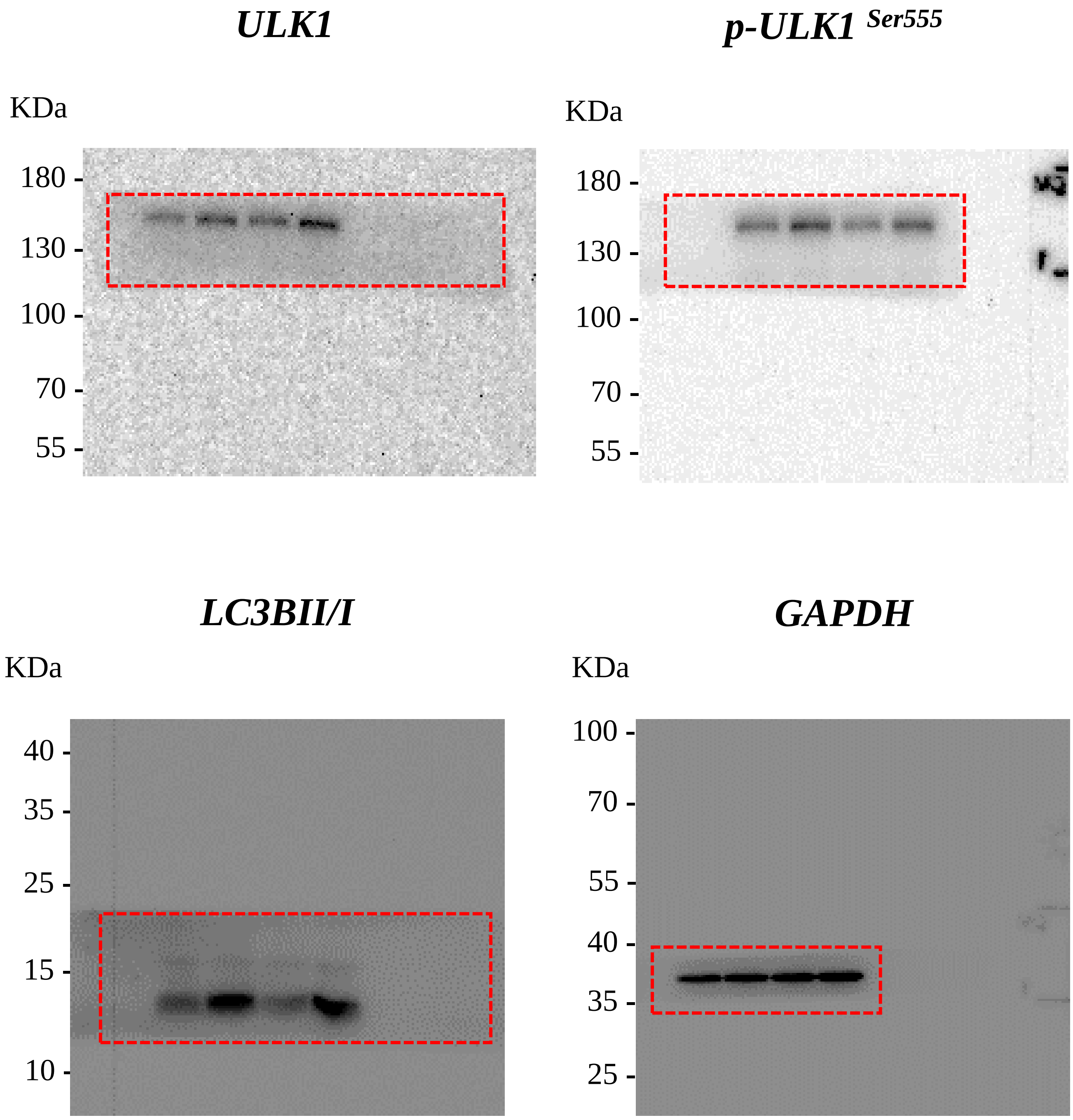

Supplementary Figure 1.

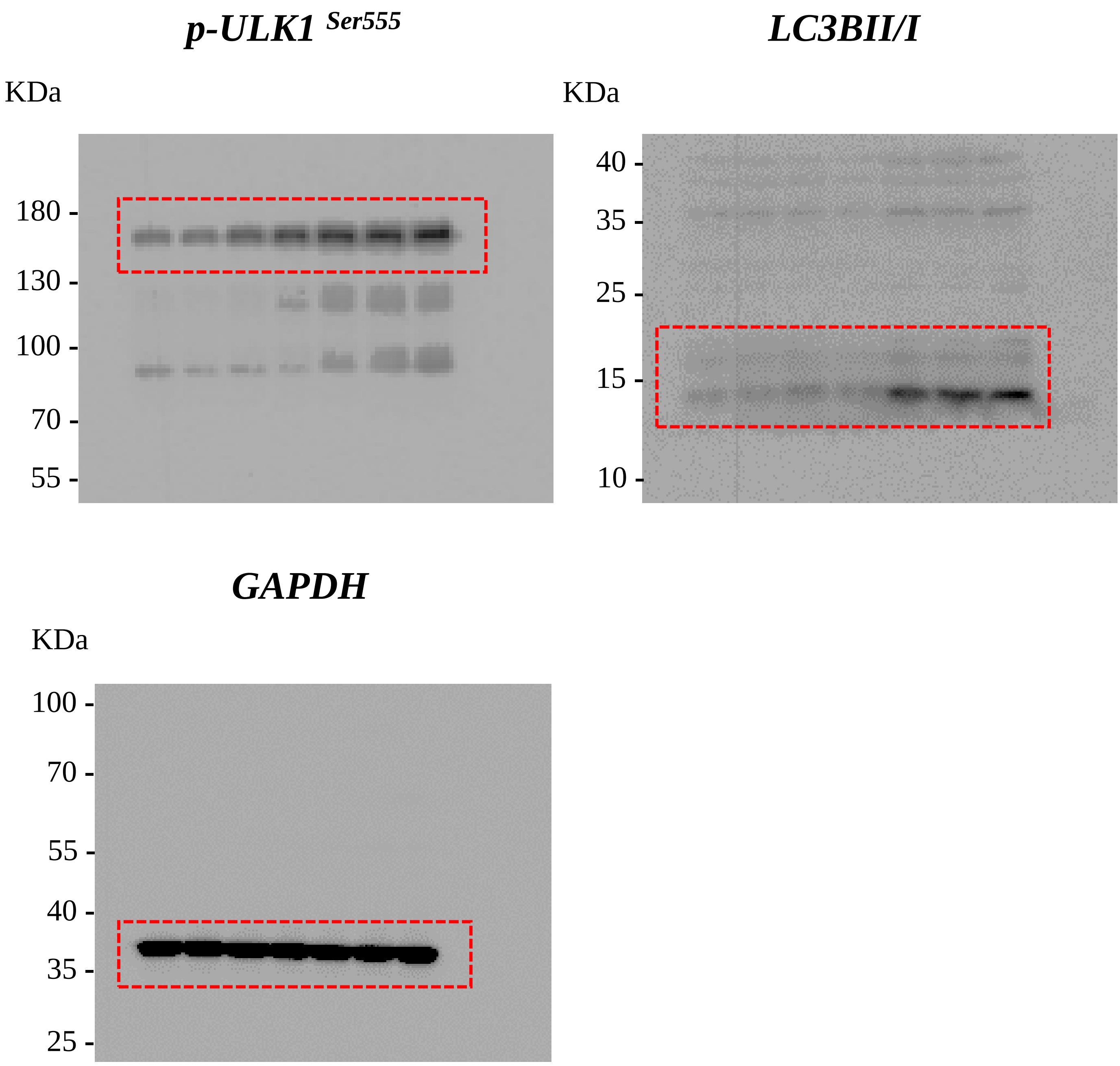

Supplementary Figure 2.

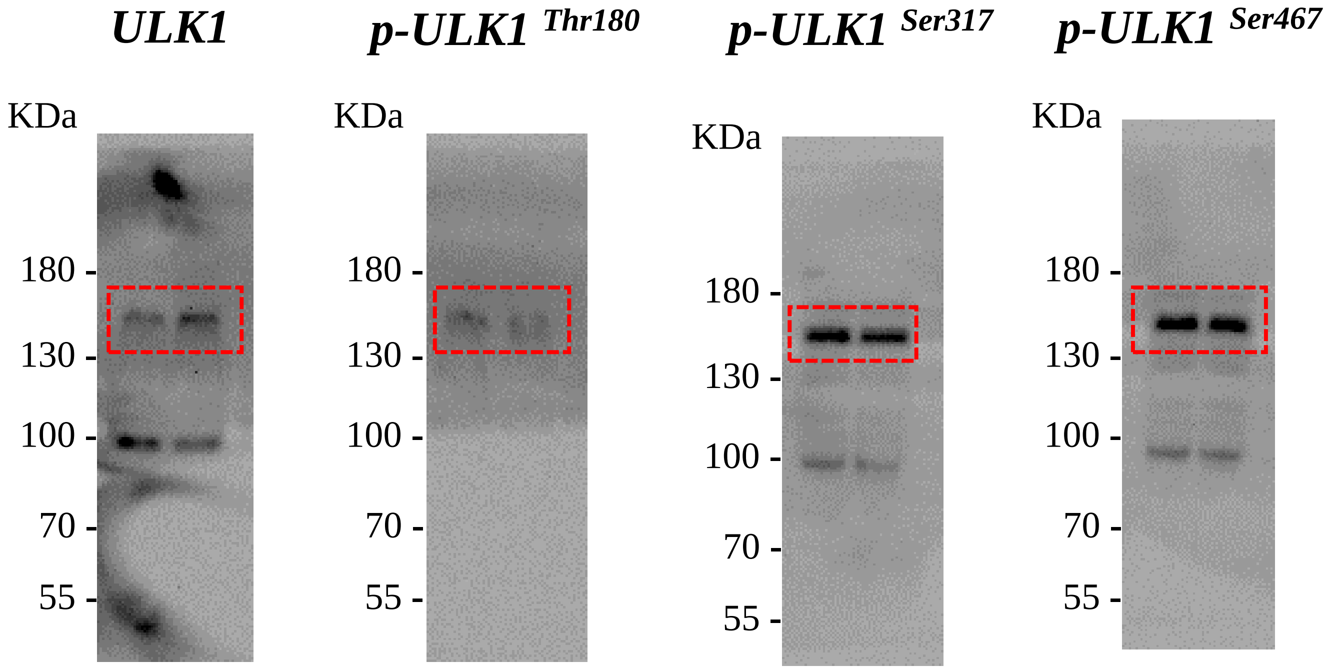

Supplementary Figure 2.

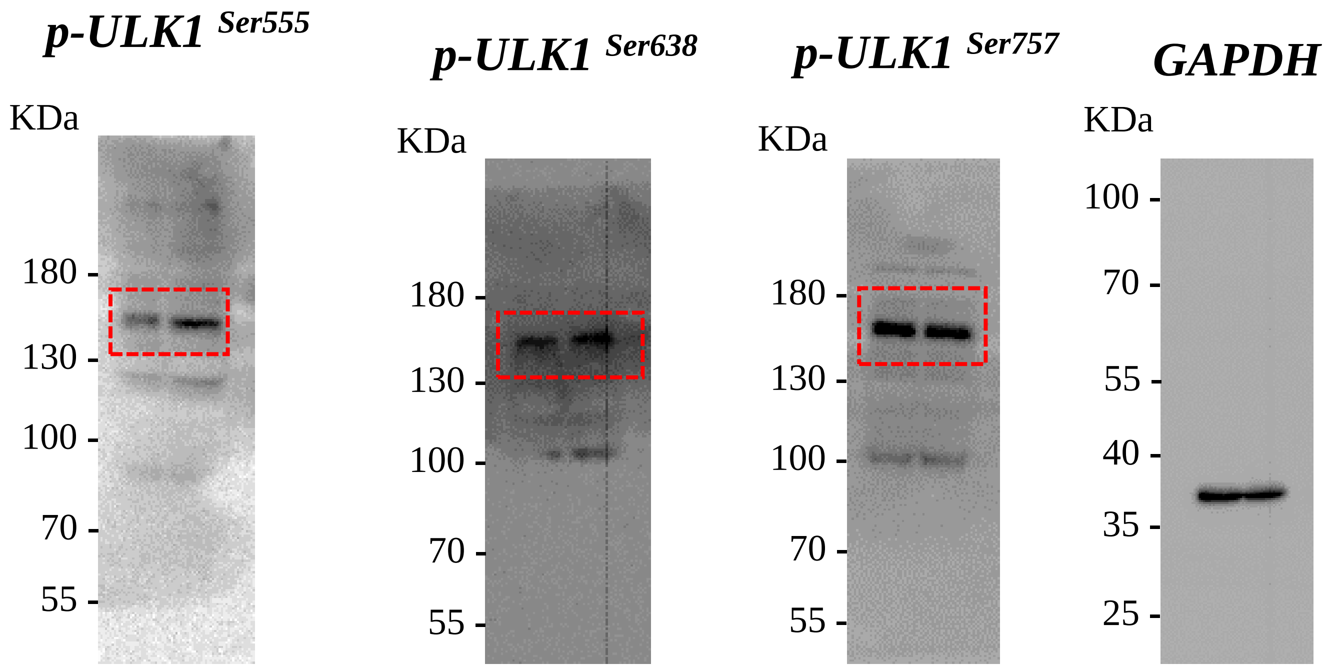

Supplement: Supplementary file 3 — Original Western blots [file 41419_2024_7084_MOESM3_ESM.pdf]
